# Supplementary material for: Division of Labor: Roles of Groucho and CtBP in Notch-Mediated Lateral Inhibition that Controls Intestinal Stem Cell Differentiation in Drosophila
Source: Stem Cell Reports. 2019 Apr 11;12(5):1007–23. doi: 10.1016/j.stemcr.2019.03.005 (PMC6523041; doi:10.1016/j.stemcr.2019.03.005)
Supplement: Document S1. Figures S1–S7 [file mmc1.pdf]

Stem Cell Reports, Volume *12*

Supplemental Information

Division of Labor: Roles of Groucho and CtBP in Notch-Mediated Lateral Inhibition that Controls Intestinal Stem Cell Differentiation in *Drosophila*

Xingting Guo, Huanwei Huang, Ziqing Yang, Tao Cai, and Rongwen Xi

Supplemental Data

Division of labor: roles of Groucho and CtBP in Notch-mediated lateral inhibition that controls intestinal stem cell differentiation in *Drosophila*

Xingting Guo[1, 2], Huanwei Huang[2], Ziqing Yang[2], Tao Cai[2] and Rongwen Xi[2, 3 *]

[1] College of Life Sciences, Beijing Normal University, Beijing 100875, China;

[2] National Institute of Biological Sciences, No. 7 Science Park Road, Zhongguancun Life Science Park, Beijing 102206, China

[3] Tsinghua Institute of Multidisciplinary Biomedical Research, Tsinghua University, Beijing, China.

* Correspondence: *xirongwen@nibs.ac.cn*

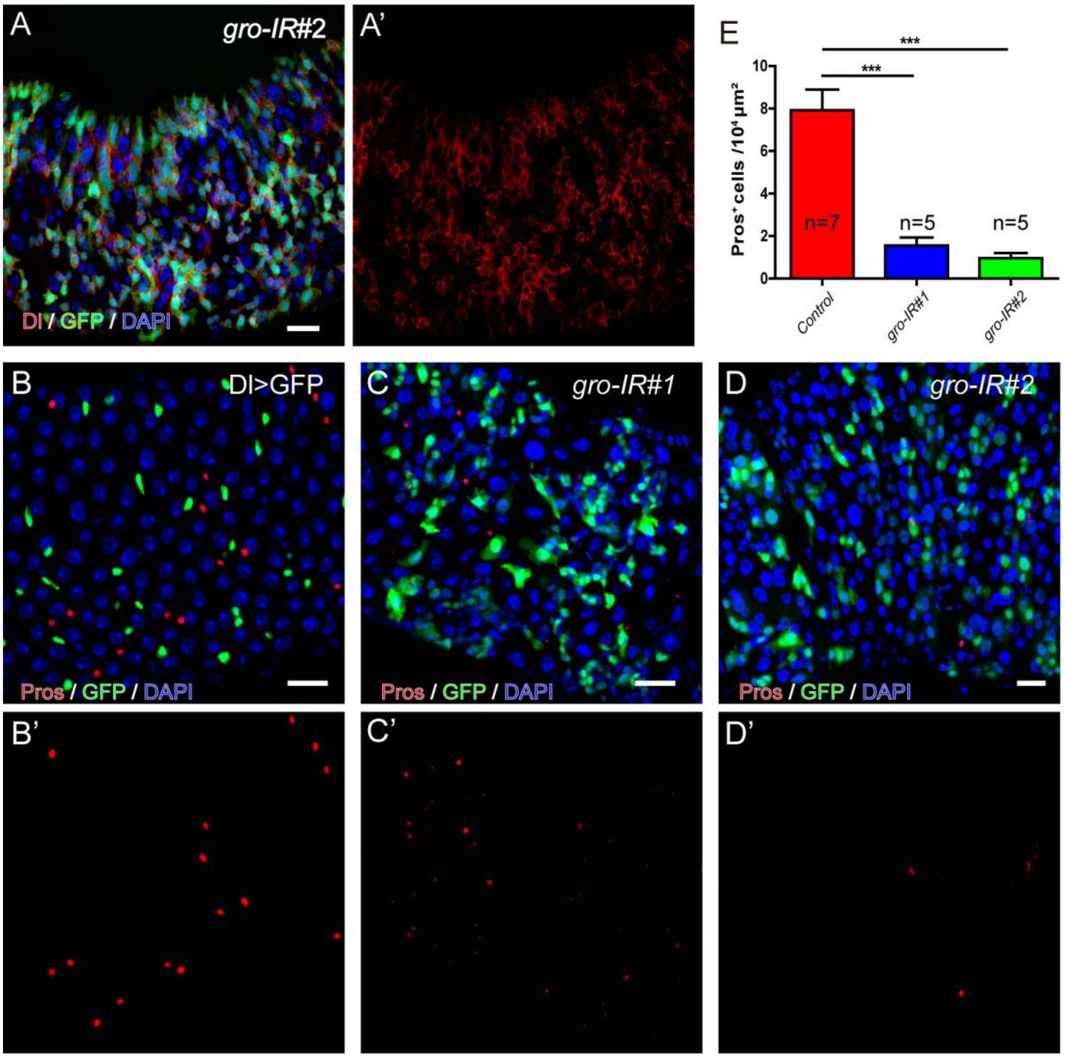

Figure S1, Related to Figure 1. Accumulation of Dl$^+$ cells and reduced EE generation following the depletion of *gro* in ISCs.

(A) Knocking down *gro* in ISCs using another RNAi line (#HMS01506) leads to similar accumulation of Dl$^+$ cells. **(B-D)** Compared to control guts, knocking down *gro* in ISCs using two independent RNAi lines causes significant reduction of Pros$^+$ cells in the intestinal epithelium. **(E)** Quantification of the density of Pros$^+$ cells in posterior midgut. Error bars indicate mean ± s.e.m. and the number of intestines calculated is labeled on the columns. ***P<0.001 (Student's t-test). Scale bars: 20

μm.

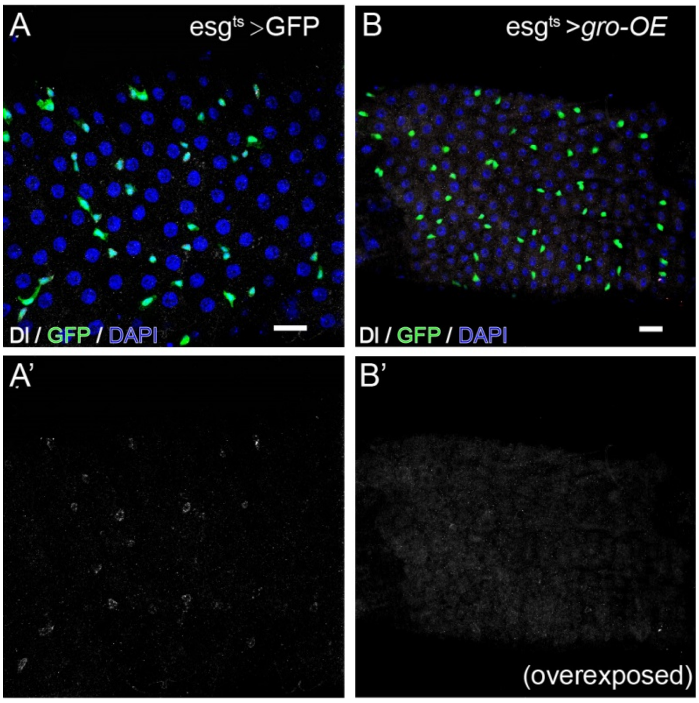

Figure S2, Related to Figure 1. *Gro* overexpression down-regulates Dl expression. Compared to control **(A)**, Ectopic expression of *gro* in esg$^+$ cells (GFP, green) rapidly down-regulates Dl expression to an undetectable level **(B)**. Note that image overexposure causes increased background signal in **B'**.

-

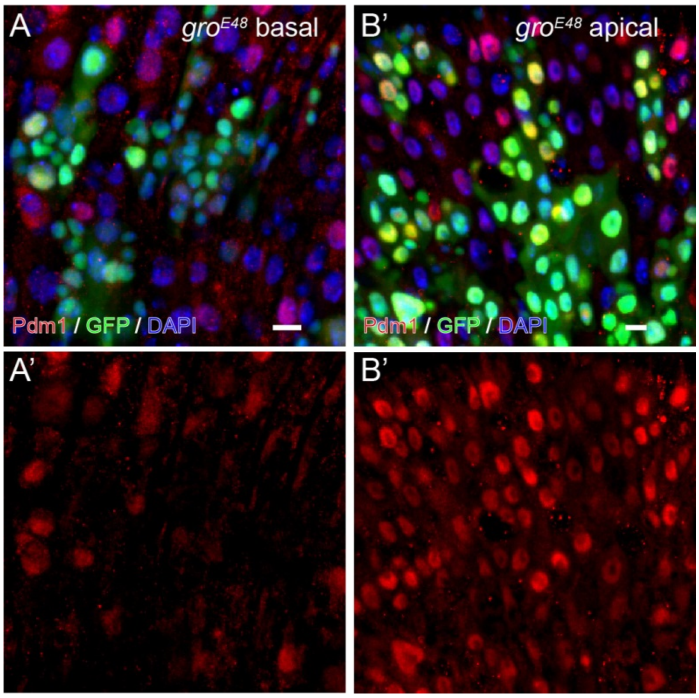

Figure S3, Related to Figure 1. The organization of Pdm1^{+} EC cells in *gro^{E48}* mutant clones.

(A-B) Pdm1^{+} polyploid cells mainly localized at apical layer (B) in the mutant clones, while most cells at basal layer (A) are Pdm1^{-} diploid cells.

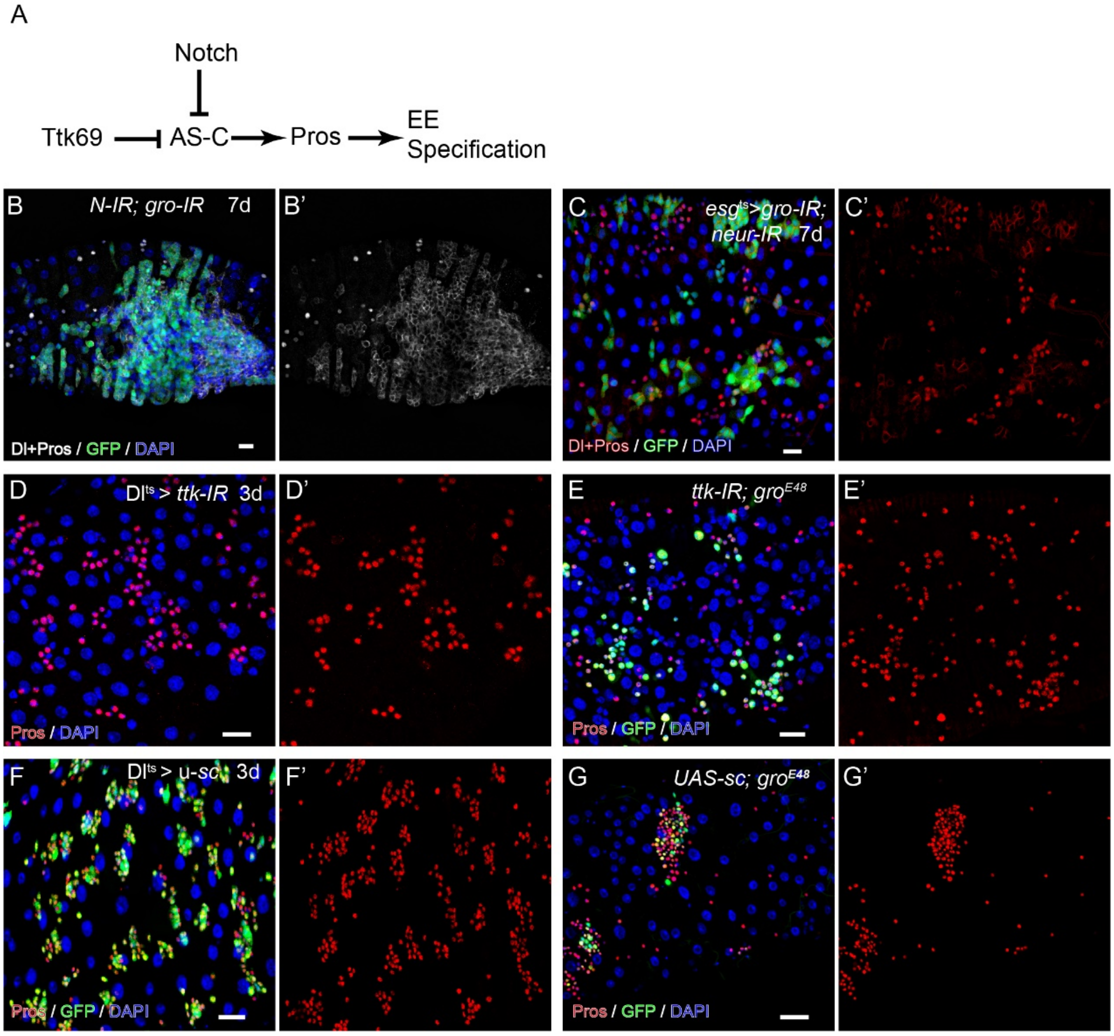

Figure S4, Related to Figure 1. *Gro* functions downstream of Notch and upstream of *ttk* and *sc* in regulating EE differentiation.

(A) A diagram of known regulatory pathways that control EE specification. **(B)** *Gro* depletion suppresses supernumerary EE generation in *Notch-RNAi* epithelium. **(C)** Loss of *gro* could not repress *neur-IR* induced supernumerary EE generation. **(D-G)** Either knocking down *ttk* or over-expressing *sc* is sufficient to cause supernumerary EE generation from ISCs, and depletion of *gro* does not prevent supernumerary EE phenotype caused by either *ttk* depletion or *sc* overexpression.

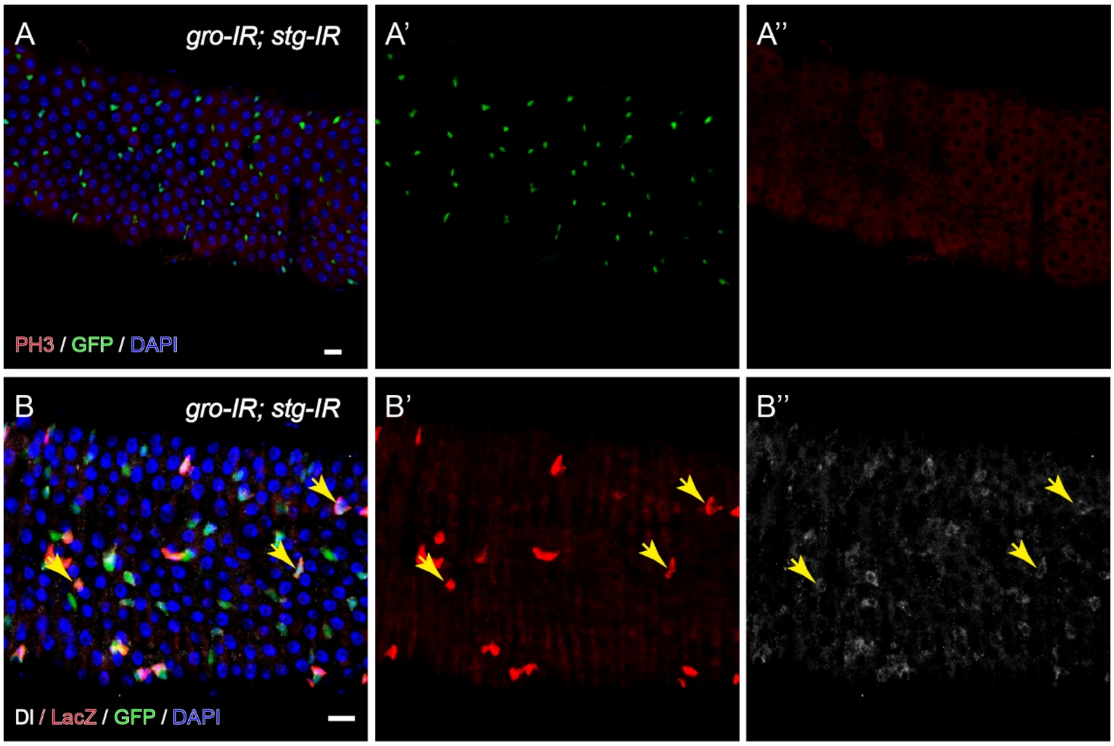

Figure S5, Related to Figure 2. Co-localization of Dl and NRE-lacZ signals could still be observed despite cell division was inhibited.

(A) Simultaneously knocking down *gro* and *stg* effectively reprerss *gro-IR* induced cell proliferation and PH3 signal is barely detected. (B) Dl^{+} NRE-$lacZ^{+}$ cells (yellow arrows) still exist in *gro-IR; stg-IR* guts.

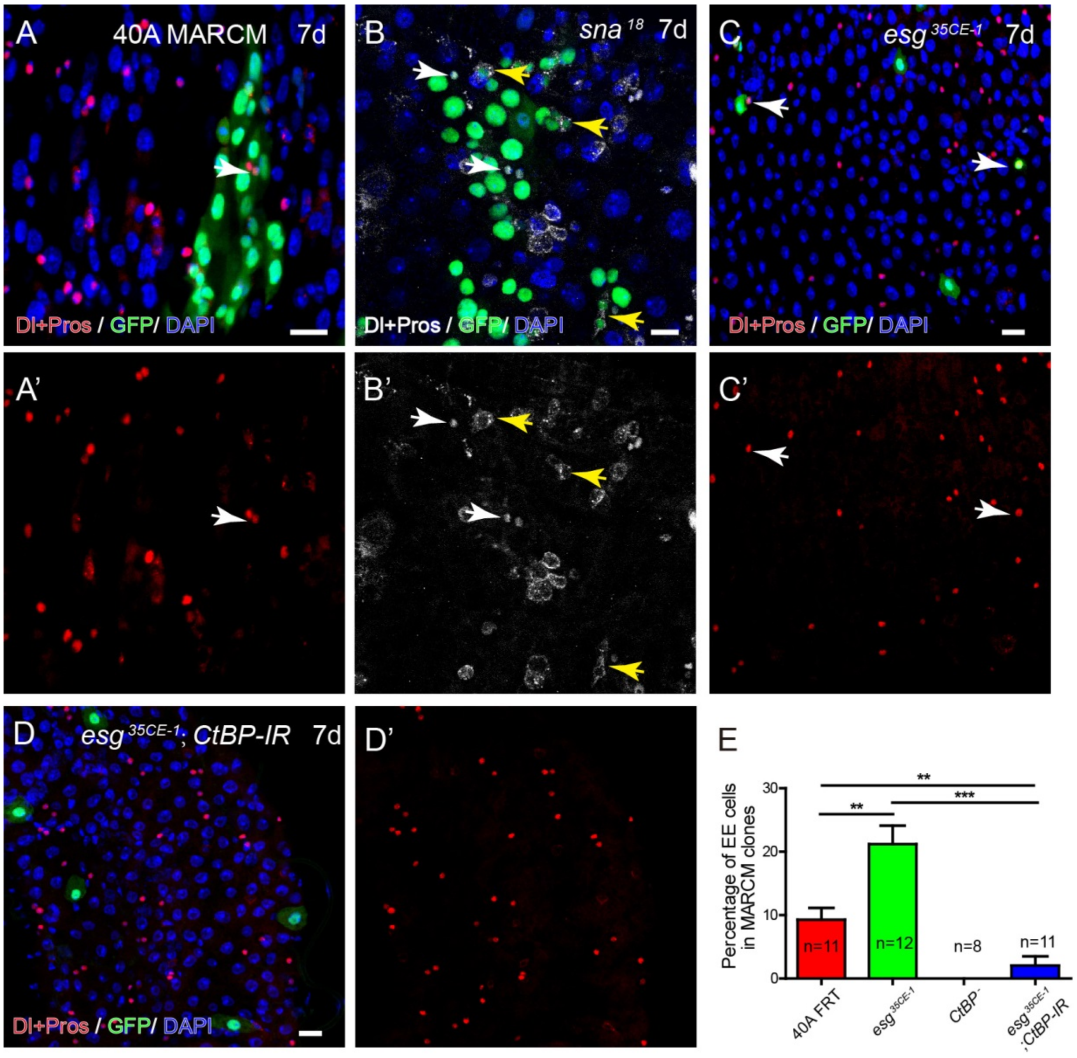

Figure S6, Related to Figure 4. Comparative analysis of *sna* and *esg* mutant clones and *esg CtBP* double mutant clones.

(A-B) Compared to control clones, depletion of *sna* exhibits no significant phenotype changes. Dl^{+} cells are indicated by yellow arrows and $Pros^{+}$ EEs by white arrows. **(C)** *esg* mutant clones do not grow and cells in the clone can different into EC or EE. **(D)** Knocking down *CtBP* in *esg* mutant clones inhibits EE differentiation. **(E)** Quantification of EE percentage in MARCM clones. Error bars indicate mean ± s.e.m. The number of intestines calculated is labeled on the columns. **P<0.01 (Student's t-test).

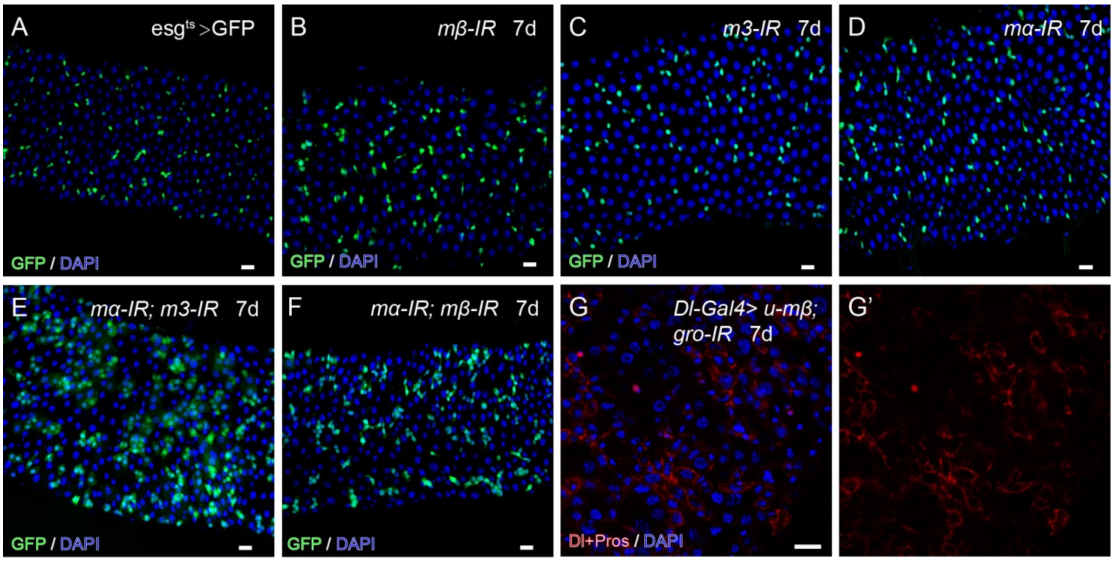

Figure S7, Related to Figure 6. *E(spl)* factors function redundantly to restrict ISC proliferation.

(A-D) Compared with control guts, separately knocking down *mα*, *m3* or *mβ* could not induce any obvious phenotype. **(E-F) S**imultaneously knocking down *mα* with *m3* or *mα* with *mβ* induced moderate accumulation of progenitor cells. **(G)** Knocking down *gro* rescues *mβ* overexpression induced ISC depletion, and causes accumulation of Dl^+ cells.
