# Supplementary material for: Division of Labor: Roles of Groucho and CtBP in Notch-Mediated Lateral Inhibition that Controls Intestinal Stem Cell Differentiation in Drosophila
Source: Stem Cell Reports. 2019 Apr 11;12(5):1007–23. doi: 10.1016/j.stemcr.2019.03.005 (PMC6523041; doi:10.1016/j.stemcr.2019.03.005)
Supplement: Document S2. Article plus Supplemental Information [file mmc2.pdf]

Stem Cell Reports
Article
OPEN ACCESS

Division of Labor: Roles of Groucho and CtBP in Notch-Mediated Lateral Inhibition that Controls Intestinal Stem Cell Differentiation in *Drosophila*

Xingting Guo,[1,2] Huanwei Huang,[2] Ziqing Yang,[2] Tao Cai,[2] and Rongwen Xi[2,3,*]
[1]College of Life Sciences, Beijing Normal University, Beijing 100875, China
[2]National Institute of Biological Sciences, No. 7 Science Park Road, Zhongguancun Life Science Park, Beijing 102206, China
[3]Tsinghua Institute of Multidisciplinary Biomedical Research, Tsinghua University, Beijing 102206, China
*Correspondence: xirongwen@nibs.ac.cn
https://doi.org/10.1016/j.stemcr.2019.03.005

SUMMARY

Intestinal stem cell (ISC) differentiation in the *Drosophila* midgut requires Delta/Notch-mediated lateral inhibition, which separates the fate of ISCs from differentiating enteroblasts (EBs). Although a canonical Notch signaling cascade is involved in the lateral inhibition, its regulation at the transcriptional level is still unclear. Here we show that the establishment of lateral inhibition between ISC-EB requires two evolutionarily conserved transcriptional co-repressors Groucho (Gro) and C-terminal binding protein (CtBP) that act differently. Gro functions in EBs with E(spl)-C proteins to suppress Delta expression, inhibit cell-cycle re-entry, and promote cell differentiation, whereas CtBP functions specifically in ISCs to mediate transcriptional repression of Su(H) targets and maintain ISC fate. Interestingly, several *E(spl)-C* genes are also expressed in ISCs that cooperate with Gro to inhibit cell proliferation. Collectively, our study demonstrates separable and cell-type-specific functions of Gro and CtBP in a lateral inhibition process that controls the proliferation and differentiation of tissue stem cells.

INTRODUCTION

Lateral inhibition, a term that originates from the field of neuroscience to describe the repression of an excited neuron toward activity of neighbors, has been used as a major mechanism to specify cell fates from initially equivalent cells during the formation of morphological patterns in advanced organisms (Gierer and Meinhardt, 1972). Lateral inhibition-mediated cell fate specification turns out to be an evolutionarily conserved mechanism, mediated primarily by Delta (Dl)-Notch signaling (Artavanis-Tsakonas et al., 1999; Lai, 2004), which regulates cell fate decisions and pattern formation in a variety of tissues throughout the animal kingdom. Well-characterized examples of lateral inhibition include its role in specifying between secretory cells and absorptive cells in the intestine, between neuronal and non-neuronal cells in the mammalian neural crest, and between bristle precursor cells and epithelial cells in *Drosophila* (Sancho et al., 2015; Simpson, 1990; Wakamatsu et al., 2000).

The transduction of Dl-Notch signaling from the cell surface to the nucleus requires the participation of a cascade of canonical signaling components as well as regulators that participate in Notch receptor glycosylation, cleavage, and transcriptional repression or activation at Notch target loci, etc. (Bray, 2006; Kopan and Ilagan, 2009). At the chromatin level, the CSL protein (also known as RBPJ, or Suppressor of Hairless [Su(H)] in *Drosophila*) acts as a bifunctional transcription factor that binds to Notch target genes. In *Drosophila*, Su(H) recruits a co-repressor Hairless, which then recruits two more global co-repressors to carry out the default repression: the C-terminal binding protein (CtBP) and Groucho (Gro). Upon Notch activation, Hairless is replaced by the intracellular domain of Notch (Nicd), which then recruits the co-activator Mastermind to promote transcriptional activation of Notch target genes (Barolo et al., 2002; Lecourtois and Schweisguth, 1995; Paroush et al., 1994; Poortinga et al., 1998; Wu et al., 2000).

The PLDLS motif-containing protein CtBP and the WD40 domain containing protein Gro belong to two distinct families of global co-repressors and are recruited to two separate motifs of the Hairless protein: CtBP is recruited to its PLDLS motif and Gro is recruited to its eh1 motif (Barolo et al., 2002; Morel et al., 2001). The combination of CtBP and Gro is required for the repressive function of Hairless in many biological processes, such as dorsoventral patterning, wing development, and peripheral nervous system development (Barolo et al., 2002; Nagel et al., 2005, 2007). However, CtBP and Gro occasionally interact independently with Hairless to regulate discrete biological processes. During certain phases of eye development, CtBP, but not Gro, is required for Hairless-mediated transcriptional repression (Nagel and Preiss, 2011). CtBP is typically thought of as a short-range co-repressor and Gro is thought of as a long-range co-repressor, so perhaps the separate or combinatory use of these two co-repressors allows for considerable flexibility to control transcriptional activity (Courey and Jia, 2001). In addition, it is known that Gro, but not CtBP, functions as a co-repressor of the major Notch transcriptional targets, the Enhancer of (spl)-complex (E(spl)-C) proteins (Paroush et al., 1994). These observations from

This is an open access article under the CC BY license (http://creativecommons.org/licenses/by/4.0/).

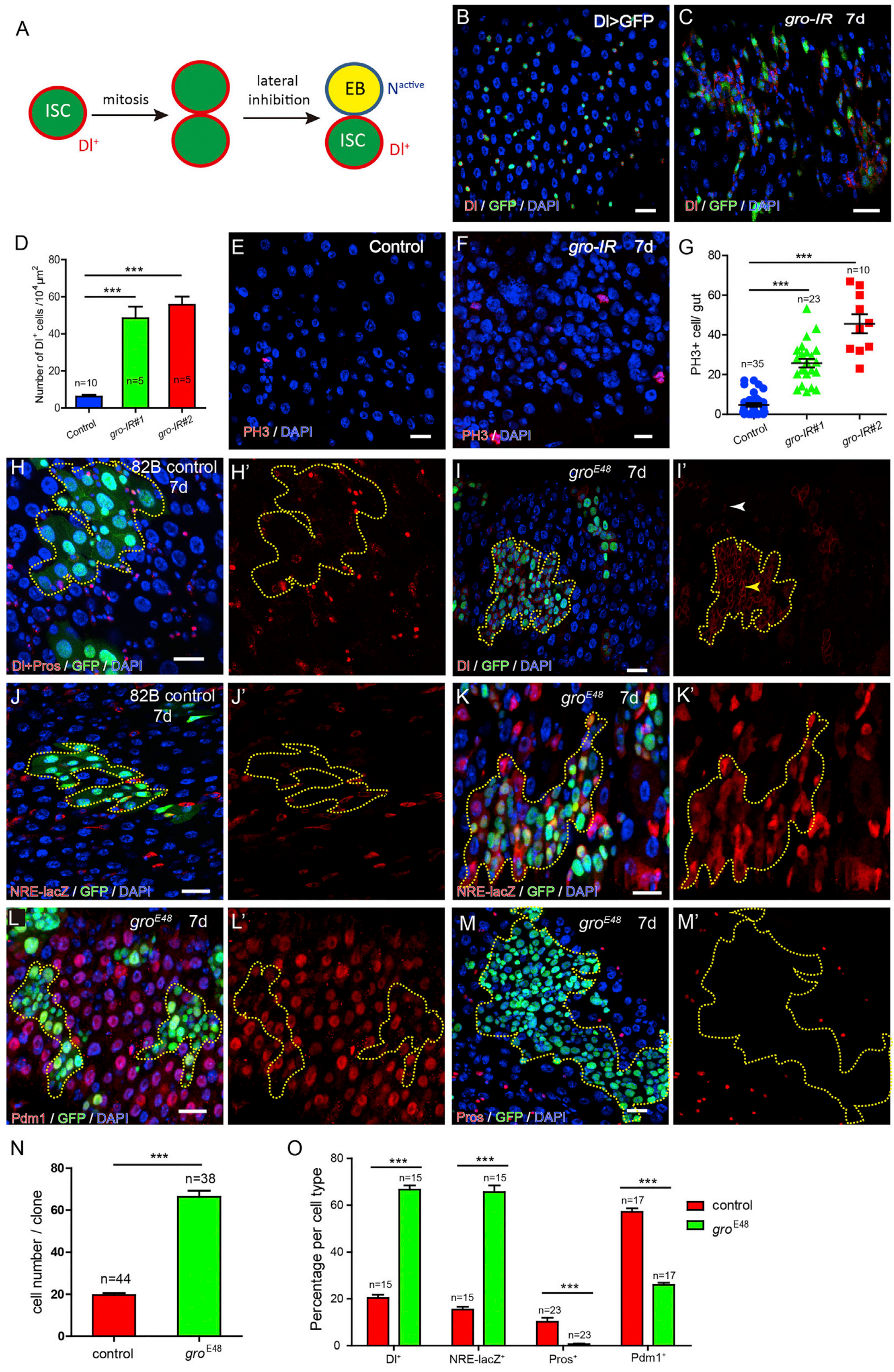

(legend on next page)

previous studies collectively show that Gro and CtBP can function together or separately with different co-factors to regulate gene transcription.

In the *Drosophila* midgut, lateral inhibition mediated by Dl-Notch signaling controls the fate of intestinal stem cell (ISC) and its immediate daughter enteroblast (EB) (Figure 1A) (Bardin et al., 2010; de Navascues et al., 2012; Micchelli and Perrimon, 2006; Ohlstein and Spradling, 2006, 2007). Dl is specifically expressed in ISCs, and typically, after each ISC division, one of the two daughters retains Dl expression and remains as an ISC, while the other daughter loses Dl expression, through the process of lateral inhibition. The Dl signal from this new ISC then triggers the activation of Notch of its sibling EB (Ohlstein and Spradling, 2007). This Notch-activated EB will eventually adopt an enterocyte fate. At a lower frequency, ISCs also divide to produce enteroendocrine cell (EE) progenitors, as a result of transient activation of a fate inducer Scute (Chen et al., 2018; Zeng and Hou, 2015). Scute then induces the expression of transcription factor Prospero (Pros), the EE fate determination factor (Bardin et al., 2010; Biteau and Jasper, 2014; Wang et al., 2015; Zeng and Hou, 2015). Although the canonical Notch signaling cascade is used in the lateral inhibition that separates ISCs from EBs, the potential engagement of co-repressors Gro and CtBP in the process has not been defined.

Here we report that depletion of Gro in ISCs in the *Drosophila* midgut causes accumulation of ISC-like cells as a result of disrupted lateral inhibition, whereas depletion of CtBP causes ISC loss because of differentiation. Our further genetic analyses have established separable functions of Gro and CtBP in lateral inhibition: CtBP specifically participates in Hairless-mediated default repression of Notch activity to maintain ISC fate, whereas Gro cooperates with E(spl)-C proteins in EBs to promote differentiation. In addition, we identified a novel role for E(spl)-C proteins in ISCs, in which they cooperate with Gro to restrict stem cell proliferation.

RESULTS

Depletion of *gro* in ISCs Causes Accumulation of Dl^+ ISC-like Cells

We performed an RNAi screen to identify new ISC regulators using fly stocks from Vienna *Drosophila* RNAi Center (VDRC) and Transgenic RNAi Project (TriP) libraries (Dietzl et al., 2007; Ni et al., 2011). These RNAi lines were crossed with flies genotyped as *Tub-Gal80^{ts}; Dl-Gal4, UAS-GFP* (for simplicity, hereafter as *Dl-Gal4^{ts}*) to perform conditional gene knockdown specifically in ISCs. ISCs, which are marked by membrane-located Dl expression, are normally sparsely distributed in the gut epithelium (Figure 1B). We found that depletion of *gro* (by VDRC line KK108953) by shifting flies to restrictive temperature for 7 days led to the formation of Dl^+ diploid cell clusters along the anterior-posterior axis of the midgut (Figures 1C and 1D). Depletion of *gro* with another independent RNAi line (HMS01506) produced a similar ISC-like cell cluster phenotype (Figure S1A). Staining with phosphor-histone 3 (PH3) antibody revealed significantly increased mitotic cells in *gro-RNAi* guts, which explains this rapid ISC-like cell accumulation phenotype (Figures 1E–1G). The epithelial density of EEs, which are marked by the nuclear localized transcription factor Pros, was significantly decreased in *gro-RNAi* guts (Figures S1B–S1E), suggesting that EE differentiation could also be affected following *gro* depletion. These observations suggest that the loss of *gro* causes blocked differentiation and continuous self-renewal of ISCs.

Gro Is Required for ISC Differentiation

Gro encodes a universal co-repressor that participates in a variety of biological processes, and is essential for viability. We generated *gro* homozygous mutant cell clones by the MARCM (mosaic analysis with a repressible cell marker) system to further investigate its function in the gut epithelium. Compared with wild-type clones of 7 days, mutant

Figure 1. *Gro* Is Required for ISC Proliferation and Differentiation

(A) An illustration of lateral inhibition-mediated asymmetric cell division of *Drosophila* ISCs. ISC, intestinal stem cell; EB, enteroblast.

(B and C) Compared with control guts (B and B′), knocking down *gro* (UAS-gro-IR no. 1) in ISCs using *Tub-gal80^{ts}; Dl-Gal4, u-GFP* for 7 days (C and C′) leads to significant accumulation of Dl^+ cells.

(D) Quantification of the Dl^+ cell density in control and *gro*-depleted posterior midgut.

(E and F) Mitotic cells in control (E) and *gro-IR* guts (F) labeled by PH3 staining.

(G) Quantification of the number of $PH3^+$ cells per gut.

(H–O) MARCM system is used to generate GFP-labeled wide type and *gro*^{E48} mutant clones (H–I′). Compared with wild-type (H) *gro* depletion leads to rapid clone expansion (I and N) and Dl^+ cell accumulation (I and O). Dl expression level is significantly higher in *gro*^{E48} mutant clones (yellow arrow) than ISCs outside the clones (white arrow) (I). The percentage of NRE-$lacZ^+$ cells is significantly increased in *gro*^{E48} mutant clones (J–K′ and O). (L and O) The percentage of ECs in total epithelial cells is reduced in *gro* mutant clones. (M and O) $Pros^+$ EE cells are rarely observed in *gro^{E48}* mutant clones.

Error bars indicate mean ± SEM. Numbers of guts/clones been calculated were labeled on the columns. ***$p < 0.001$ (Student's t test). Scale bars, 20 μm.

clones homozygous for *gro*^{E48}, a loss-of-function allele of *gro* (Jennings et al., 2006), grew much more rapidly and form multilayers. By quantification, each mutant clone contained significantly more cells compared with the wild-type clone (Figure 1N). In addition, the mutant clones contained significantly more Dl^{+} cells, consisting of more than 60% of the mutant cells, and the level of Dl expression in these mutant cells (yellow arrow) was generally higher than that in wild-type ISCs (white arrow) outside the mutant clones (Figures 1H, 1I, and 1O). Consistent with a role for Gro in suppressing Dl expression, transient overexpression of *gro* using a *UAS-gro* transgene by esg-Gal4^{ts}, an ISC and EB cell driver, effectively shut down Dl expression in all ISCs (Figure S2).

A Notch activation reporter Gbe-Su(H)m8-lacZ (or NRE-lacZ), which uses three copies of Grh binding sites and two copies of Su(H) binding sites from *E(spl)m8* as its enhancer (Furriols and Bray, 2001), normally marks committed progenitor cells for enterocyte (EC), and transcription factors Pdm1 and Pros marks EC and EE cells, respectively. By immunostaining with these cell fate markers, we found that the percentage of NRE-lacZ^{+} cells was greatly increased in *gro*^{E48} mutant clones (Figures 1J, 1K, and 1O). Pdm1^{+} cells were still present within the mutant clones, although their percentage was decreased and they typically formed a single layer lining the apical surface of the mutant clones (Figures 1L, 1O, and S3). Strikingly, Pros^{+} cells were virtually absent in the mutant clones (Figures 1M and 1O). Therefore, loss of *gro* causes accumulation of ISC-like cells and failure of EE generation, but EC differentiation still occurs. These data demonstrate that *gro* is required for efficient ISC differentiation and EE generation.

Previous studies have demonstrated that Notch and Ttk69 act in parallel to suppress AS-C genes *scute* (*sc*) and *asense* (*as*), and the activation of *sc* is sufficient to induce Pros expression and consequently EE differentiation. As a result, depletion of Notch or disruption of the Ttk69-Phyl-AS-C regulatory cassette in ISCs is sufficient to induce excessive EE generation, leading to EE tumors (Chen et al., 2018; Micchelli and Perrimon, 2006; Ohlstein and Spradling, 2006; Wang et al., 2015; Yin and Xi, 2018). Our epistasis analysis indicates that *gro* functions upstream of the Ttk69-Phyl-AS-C cassette in regulating EE differentiation, because depleting *ttk* or overexpressing *sc* causes continuous EE generation, even when *gro* is depleted (Figures S4D–S4G). The genetic relationship between *gro* and *Notch* seems to be complicated. Co-depleting *gro* with two key component of Notch signaling, *Notch* or *neuralized* (*neur*), exhibits distinct outcomes on EE differentiation. Co-depleting *gro* with *Notch* caused formation of ISC-like tumors but not EE tumors in the gut (Figure S4B). However, co-depleting *gro* with *neur,* which encodes an E3 liganse that promotes Notch signaling by endocytosis-dependent activation of the ligands (Lai et al., 2001; Pavlopoulos et al., 2001), caused both ISC-like and EE tumors in the gut (Figure S4C), similar to those caused by *E(spl)-C* and *neur* double mutations reported previously (Bardin et al., 2010). What causes this phenotypic difference is unclear, but it is possible that this may be due to Notch-independent functions of Neur (Chanet and Schweisguth, 2012). In addition, there might be a non-autonomous role of neur in activating Dl, or there might be a subtle role for Serrate, the other Notch ligand, in the process.

Lateral Inhibition between the ISC Daughters Is Disrupted in the Absence of *gro*

The increased NRE-lacZ^{+} cells in *gro* mutant clones led us to examine whether the lateral inhibition process mediated by Dl-Notch signaling between the two newly formed ISC daughters is compromised. In normal midgut, each ISC and its immediate daughter EB are frequently in juxtaposed with each other, forming a pair (Ohlstein and Spradling, 2007). In each pair, Dl is specifically expressed in the ISC, NRE-lacZ is turned on specifically in the EB, and Dl and NRE-lacZ are never co-expressed in the same cell (Figure 2A, yellow and green arrows). Interestingly, conditional depletion of *gro* using the *esg-gal4* driver caused many of these pairs to have altered expression of Dl and NRE-lacZ. Frequently, Dl expression could be found in both cells in the pair, while one of the pair expressed NRE-lacZ. This results in the co-expression of Dl and NRE-lacZ in one cell of the pair, presumably the EB cell (Figure 2B). Similarly, in *gro*^{E48} mutant clones, many cells co-expressed Dl and NRE-lacZ. Dl^{+} NRE-lacZ^{-} cells, the presumptively ISCs, were also found to be intermingled in between Dl^{+} NRE-lacZ^{+} cells, the presumptively EBs (Figure 2C). The appearance of Dl^{+} NRE-lacZ^{+} cells is likely due to disrupted lateral inhibition, but it is possible that enhanced cell-cycle kinetics following *gro* depletion might also contribute to it. To minimize this effect, we simultaneously knocked down *gro* and *string* (*stg*), an essential trigger for mitosis. This caused loss of mitotic activity in the gut epithelium, but many Dl^{+} NRE-lacZ^{+} cells still appeared (Figure S5). Collectively, these observations suggest that loss of *gro* seems to cause retained Dl expression in the daughter EB cells, while *Hairless*-mediated transcriptional repression remained effective in ISCs.

To further investigate the transcriptional changes in *gro*-depleted cells, we sorted Dl > GFP^{+} cells in *gro-RNAi* and control guts, and performed RNA sequencing (RNA-seq) analysis. The results revealed that many known Notch target genes, including *hey*, *vg*, and an array of *E(spl)-C* genes, are significantly upregulated in *gro-RNAi* guts (Figure 2G, red spots), indicating ectopic activation of Notch

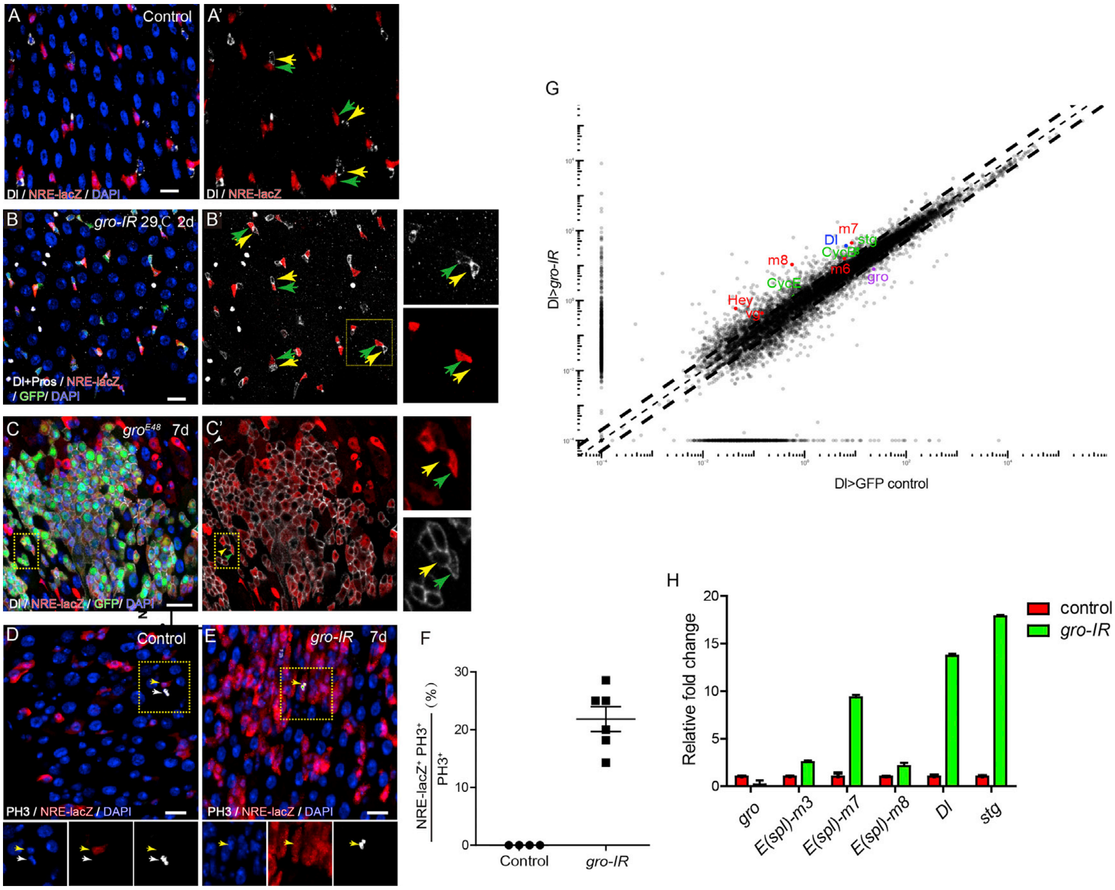

Figure 2. *Gro* Depletion Disrupts Delta/Notch-Mediated Lateral Inhibition between the Two ISC Daughters

(A) In normal guts, Dl and NRE-lacZ never co-localize in the same cell (yellow and green arrows).

(B) Knocking down *gro* in progenitor cells using esg-Gal4 leads to co-localization of Dl and NRE-lacZ signals (green arrows).

(C) Dl^{+} NRE-$lacZ^{-}$ cells (yellow arrow) and Dl^{+} NRE-$lacZ^{+}$ cells (green arrow) are found in gro^{E48} mutant clones.

(D) PH3 signal (white arrows) is detected only in NRE-$lacZ^{-}$ cells in normal guts.

(E) Co-localization of PH3 and NRE-lacZ after *gro* depletion (yellow arrows) in progenitor cells.

(F) Quantification of the percentage of $PH3^{+}$ NRE-$lacZ^{+}$ cells in total $PH3^{+}$ cells of control and *gro*-depleted guts.

(G) Transcriptional changes of FACS-sorted Dl-GFP cells in control and *gro-RNAi* guts. After *gro* depletion, many known Notch target genes are upregulated (red spots); Dl (blue spot) and several cell-cycle genes (green spots) are also upregulated.

(H) qRT-PCR validation of transcriptional changes caused by *gro* depletion (results from three independent biological replicates).

signaling in *gro*-depleted Dl^{+} cells. Interestingly, the expression level of Dl was also increased by approximately 6-fold (Figure 2G, blue spot), and several cell-cycle-related genes, such as *stg* and *cycE*, were also upregulated (Figure 2G, green spots). RT-PCR analysis further validated some of these expression changes (Figure 2H). Interestingly, the NRE-$lacZ^{+}$ cells in *gro-RNAi* gut were able to divide, as revealed by PH3 staining, although normally the NRE-$lacZ^{+}$ cells are post-mitotic cells (Figures 2D–2F). These observations indicate that depletion of *gro* causes retained expression of Dl in the presumptive EBs and allows them to re-enter cell cycle.

To directly test the function of *gro* in EBs, we depleted *gro* specifically in EBs using the NRE-Gal4 driver. Normally in EBs, Notch signaling is activated, which further represses Dl expression and promotes cell differentiation. We found that on depletion of *gro* in EBs, Dl expression was re-appeared (Figures 3A and 3B), and these *gro*-depleted EBs were also able to re-enter cell cycle (Figures 3C and 3D). A cell lineage-tracing experiment using the NRE-GAL4, UAS-flp and flp-out cassette system (Figure 3E) demonstrated that *gro*-depleted EBs were able to give rise to small cell clusters expressing both the lineage marker LacZ and Dl, while normal EBs only gives rise to polyploid ECs (Figures 3F and 3G).

Collectively, these data demonstrate that *gro* is necessary to establish Dl-Notch-mediated lateral inhibition between the two immediate ISC daughters (the presumptive ISC

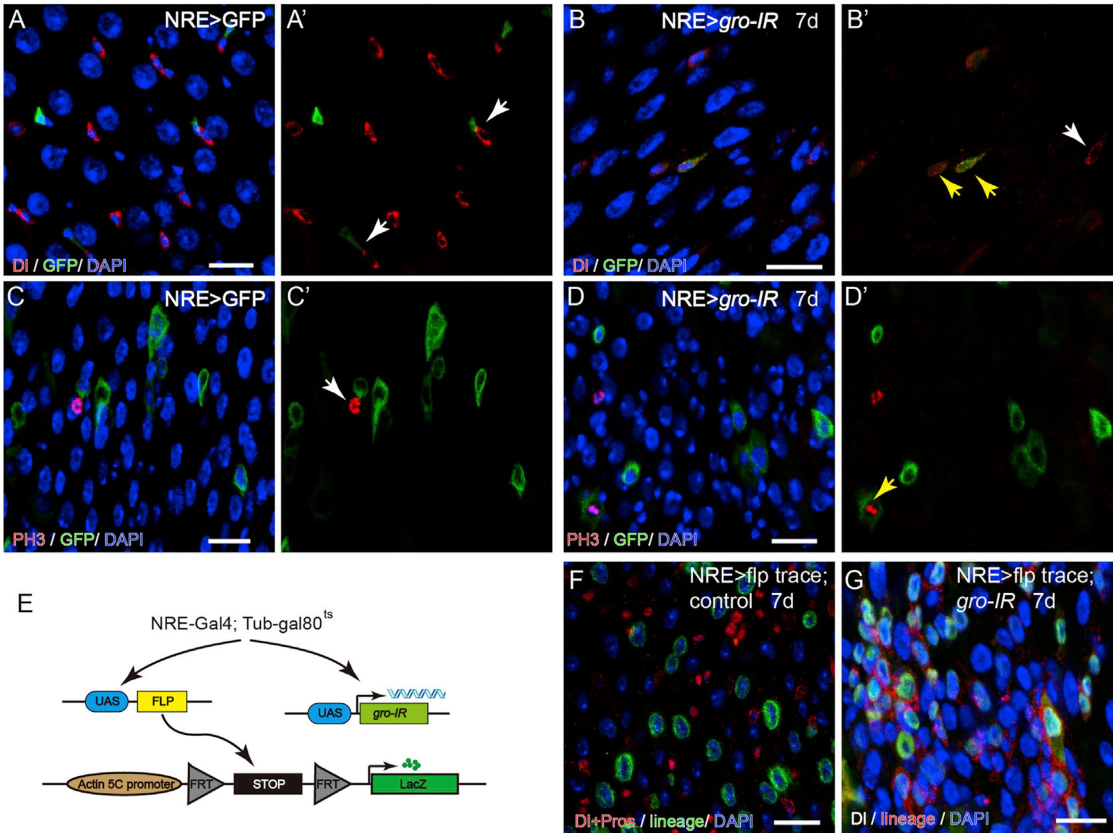

Figure 3. Depletion of *gro* in EBs Causes Cell-Cycle Re-entry and Blocked Differentiation

(A and B) Compared with control guts (A), knocking down *gro* in EBs (B) using NRE-Gal4^{ts} leads to Dl expression in EBs (marked by GFP, green).

(C and D) No PH3 signals could be detected in GFP^{+} EB cells in normal guts (C), while *gro* depletion in EBs allows EB to re-enter the cell cycle, as indicated by co-localization of NRE-GFP and PH3 signals (D).

(E) A schematic of cell lineage-tracing strategy.

(F) In wild-type guts, NRE-Gal4^{+} cells mostly give rise to polyploid EC cells.

(G) Following *gro* depletion, NRE-Gal4^{+} cells give rise to clusters of diploid cell progeny and many of them express Dl.

and the presumptive EB) by preventing Dl expression and mitotic re-entry in the presumptive EB. Interestingly, as NRE-lacZ is not activated in ISCs following *gro* depletion, *gro* is not required for Su(H)/Hairless-mediated transcriptional repression in ISCs, indicating that other co-repressor(s) could participate in the process.

CtBP Is Essential to Repress Notch Signaling Activation in ISCs

Another candidate co-repressor potentially involved in this process is CtBP, as both CtBP and Gro interact with Hairless and are found to collectively participate in Hairless-mediated transcriptional repression in many biological processes. We transiently depleted *CtBP* in progenitor cells by RNAi, and examined the effect on NRE-lacZ expression in ISCs and EBs. Strikingly, transient depletion of *CtBP* rapidly abolished Dl expression in progenitor cells and rendered the majority of these cells to express NRE-lacZ (Figure 4A). This is very different from the effect by *gro-IR*, but is similar to the effect by Nicd overexpression (Figure 4B). Therefore, Notch signaling is ectopically activated in ISCs following *CtBP* depletion. To further confirm that depletion of *CtBP* is sufficient to induce transcriptional activation of Notch signaling without Notch receptor activation, we co-depleted *Notch* and *CtBP* in ISCs and EBs and examined the effect on NRE-lacZ expression. As a control, either depletion of *Notch* or co-depletion of *Notch* and *gro* failed to activate NRE-lacZ expression in the ISC-like tumor cells (Figures 4C and 4D). However, co-depletion of *Notch* and *CtBP* prevented ISC proliferation and caused virtually all ISCs to ectopically expression NRE-lacZ (Figure 4E).

It is known that Hairless interacts with CtBP through its C-terminal domain. Consistent with our hypothesis that

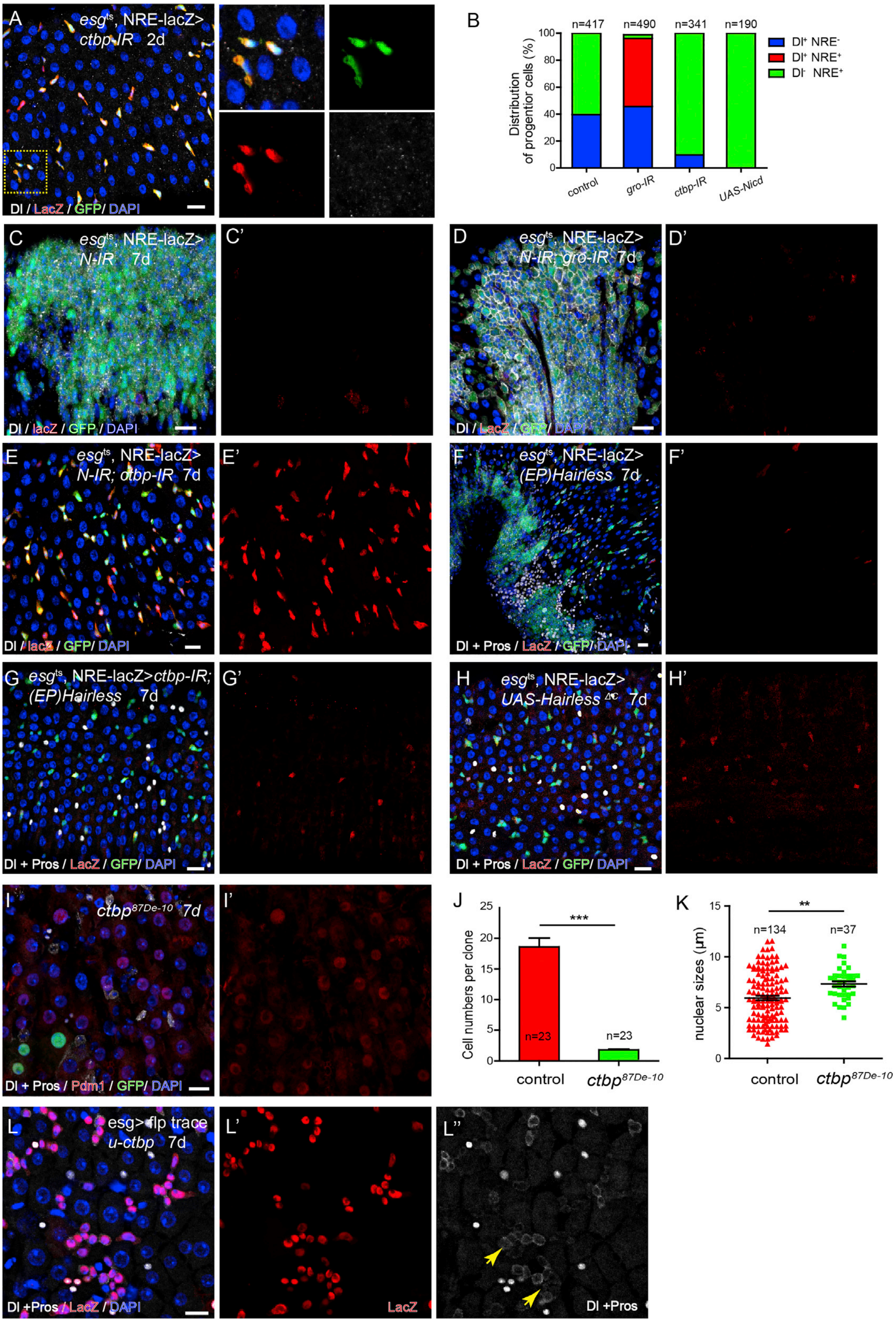

(legend on next page)

Hairless utilizes CtBP as cofactor to exert the default repression, we found that depletion of *CtBP* in esg^{+} cells completely suppressed Hairless overexpression-induced ISC-like and EE-like tumors, and allowed NRE-lacZ expression in all of these esg^{+} cells (Figures 4F and 4G). In addition, in contrast to the full-length Hairless, overexpression of a C-terminal truncated form of Hairless ($H^{\Delta C}$) failed to induce any ISC- or EE-like cell accumulation in the epithelium, and failed to suppress NRE-lacZ expression (Figure 4H). Collectively, these data demonstrate that Hairless only uses CtBP as a cofactor to exert the default repression in ISCs.

To further understand the fate of *CtBP* mutant ISCs, we induced *CtBP* $^{87De-10}$ (a loss-of-function allele) homozygous mutant clones using the MARCM system, and examined the clones after 7 days. The mutant clones typically contained only one or two polyploid cells, positive for Pdm1 (Figures 4I–4K), suggesting that loss of *CtBP* causes ISCs to rapidly differentiate into ECs. Interestingly, continuous overexpression of *CtBP* was also able to hamper ISC differentiation, as revealed by the fact that the cell lineage-tracing marker that *CtBP* overexpressed ISCs gave rise to many Dl^{+} cells that were clustered together (Figure 4L, arrows), although CtBP overexpression did not completely block EC differentiation.

It has been reported that CtBP also serves as a co-repressor for Snail family transcription factors Esg and Snail to regulate gene expression (Nibu et al., 1998; Voog et al., 2014), and both Esg and Snail have been implicated in the regulation of ISC maintenance and/or proliferation (Dutta et al., 2015; Korzelius et al., 2014; Loza-Coll et al., 2014). However, loss of *esg* causes ISCs to differentiate into both ECs and EEs, with an increased preference for EEs (Figure S6C) (Li et al., 2017; Loza-Coll et al., 2014), a phenotype that is different from the loss of *CtBP* (Figures 4I–4K), implying that CtBP mainly interacts with Hairless but not Esg to regulate ISC differentiation. We found that EE generation was disrupted in *CtBP* and *esg* double-depleted clones, similar to *CtBP*-depleted clones (Figure S6D). This further supports an Esg-independent function of CtBP in regulating ISC differentiation. We also re-examined the function of *sna* by clonal analysis. We did not observe any obvious phenotype in *sna*^{18} (a loss-of-function allele) mutant ISC clones. The clone size was comparable with the wild-type controls, and the cell-type composition in the mutant clones also appeared normal (Figures S6A and S6B). Therefore, unlike Esg, Snail is not a critical factor for ISC proliferation and differentiation.

Gro Is Necessary for E(spl)-C-Mediated Repression of Dl Expression and Cell Division

In the midgut, the *E(spl)-C* genes are major targets of Notch signaling that promote ISC differentiation. As Gro can serve as a co-repressor of E(spl)-C proteins to carry out transcriptional repression, it is reasonable to speculate that the disrupted differentiation phenotype following *gro* depletion could be due to compromised activity of E(spl)-C proteins. To test this possibility, we first asked whether *gro* is required for Notch activation-induced ISC differentiation. Ectopic activation of Notch pathway by overexpressing intracellular domain of Notch (Nicd) in ISCs was able to rapidly repress Dl expression, and eventually induce their loss by differentiation into ECs (Figures 5A–5B′), as previously observed (Micchelli and Perrimon, 2006; Wang et al., 2015). However, simultaneous depletion of *gro* during *Nicd* overexpression was able to effectively prevent ISC differentiation, and, on the contrary, extra Dl^{+} ISC-like cells were produced (Figure 5C). This is similar to the phenotype caused by *gro* depletion alone, thus suggesting that *gro* genetically acts downstream of *Nicd*. Next, we asked whether *gro* is required for ISC differentiation induced by *E(spl)-C* genes. Clonal overexpression of *E(spl)m7* was sufficient to induce ISC loss and EC differentiation (Figure 5D). In contrast, overexpression of *E(spl)m7* in *gro* mutant clones failed to

Figure 4. CtBP Is Essential to Repress Notch Signaling Activation in ISCs

(A and A′) Knocking down *CtBP* in progenitor cells (genotyped as esg-Gal4, u-GFP; NRE-lacZ, Tub-Gal80^{ts}, or esg^{ts}; NRE-lacZ for simplicity) leads to Notch activation in the paired progenitor cells (yellow arrow).

(B) Quantification of the percentage of Dl^{+} NRE^{-}, Dl^{+} NRE^{+}, and Dl^{-} NRE^{+} cells among progenitors after treatment for 7 days.

(C and D) Knocking down *Notch* disrupts activation of NRE-lacZ reporter (C), and *Gro* depletion fails to turn on NRE-lacZ expression in *Notch-IR* guts (D).

(E) *Notch* depletion-induced tumor is suppressed in the absence of *CtBP*, and NRE-lacZ is generally activated in the mutant progenitor cells.

(F and G) Overexpressing *Hairless* blocks Notch activation and induces tumorigenesis (F), and co-depleting *CtBP* efficiently suppresses tumorigenesis and allows activation of NRE-lacZ in progenitor cells (G).

(H) Overexpressing a truncated form of Hairless ($H^{\Delta C}$) fails to suppress NRE-lacZ expression and induce tumorigenesis.

(I) ISC is absent in *CtBP* mutant clones and most of the mutant clones consist of only one or two Pdm1^{+} polyploid cells.

(J–L) Quantifications of cell number (J) and nuclear size (K) in control and *CtBP* mutant clones. Error bars indicate mean ± SEM, ***$p < 0.001$ (Student's t test). Scale bars, 20 μm. (L) Lineage tracing of esg^{+} cells with *CtBP* overexpression. Dl^{+} cell clusters were observed in the lineage (yellow arrow).

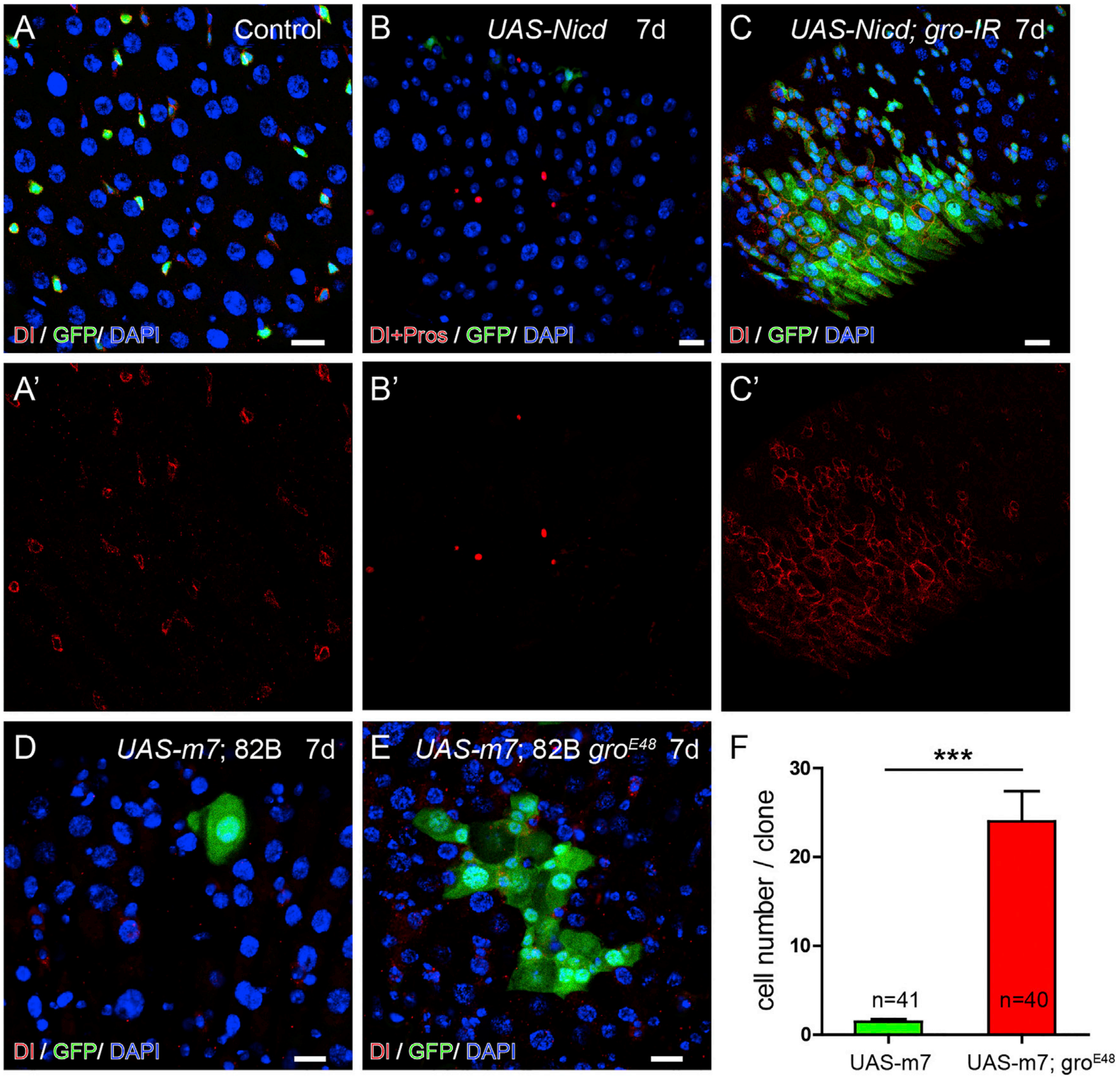

Figure 5. ***Gro*** **Is Necessary for E(spl)-C-Mediated Repression of Dl Expression and Cell Division**

(A–B′) Overexpressing Nicd using Dl-Gal4 effectively depletes Dl$^+$ ISCs (B and B′), compared with the control guts (A and A′).

(C and C′) Knocking down *gro* prevents Nicd-induced ISC depletion. To the contrary, it leads to accumulation of Dl$^+$ cells.

(D) Clonal overexpression of *m7* alone causes ISC loss and differentiation into ECs.

(E) Overexpression of *m7* in *gro*^{E48} mutant clones fails to deplete ISCs, as the clones are able to grow into large patches.

(F) Quantification of MARCM clone sizes in *UAS-m7* and *UAS-m7; gro*^{E48} guts, n = 40–50 clones.

Error bars indicate mean ± SEM, ***p < 0.001 (Student's t test). Scale bars, 20 μm.

deplete ISCs, as the mutant clones were still able to grow into a large number of cells (Figures 5E and 5F). It is worth mention that the phenotypes associated with *gro* mutant clones, including excessive Dl$^+$ cells and absence of EEs, are reminiscent to *P[gro+] Df*(3R) *gro32.2* mutant clones, in which the entire *E(spl)-C* genes are removed (Bardin et al., 2010). Collectively, these data are consistent with the idea that Gro functions in EBs as a co-repressor for E(spl)-C proteins to repress Dl expression and facilitate cell differentiation.

Gro Mediates a Baseline Notch Activity in ISCs to Inhibit Cell Proliferation

In addition to disrupted lateral inhibition, depleting *gro* in ISCs also leads to increased mitosis in the midgut. This could be an indirect effect caused by accumulation of ISC-like cells that are proliferative. Alternatively, *gro* could have a negative role in regulating ISC proliferation. To study whether *gro* has a separate role in cell proliferation, in addition to the role in lateral inhibition, we clonally overexpressed *gro* using the MARCM system and found

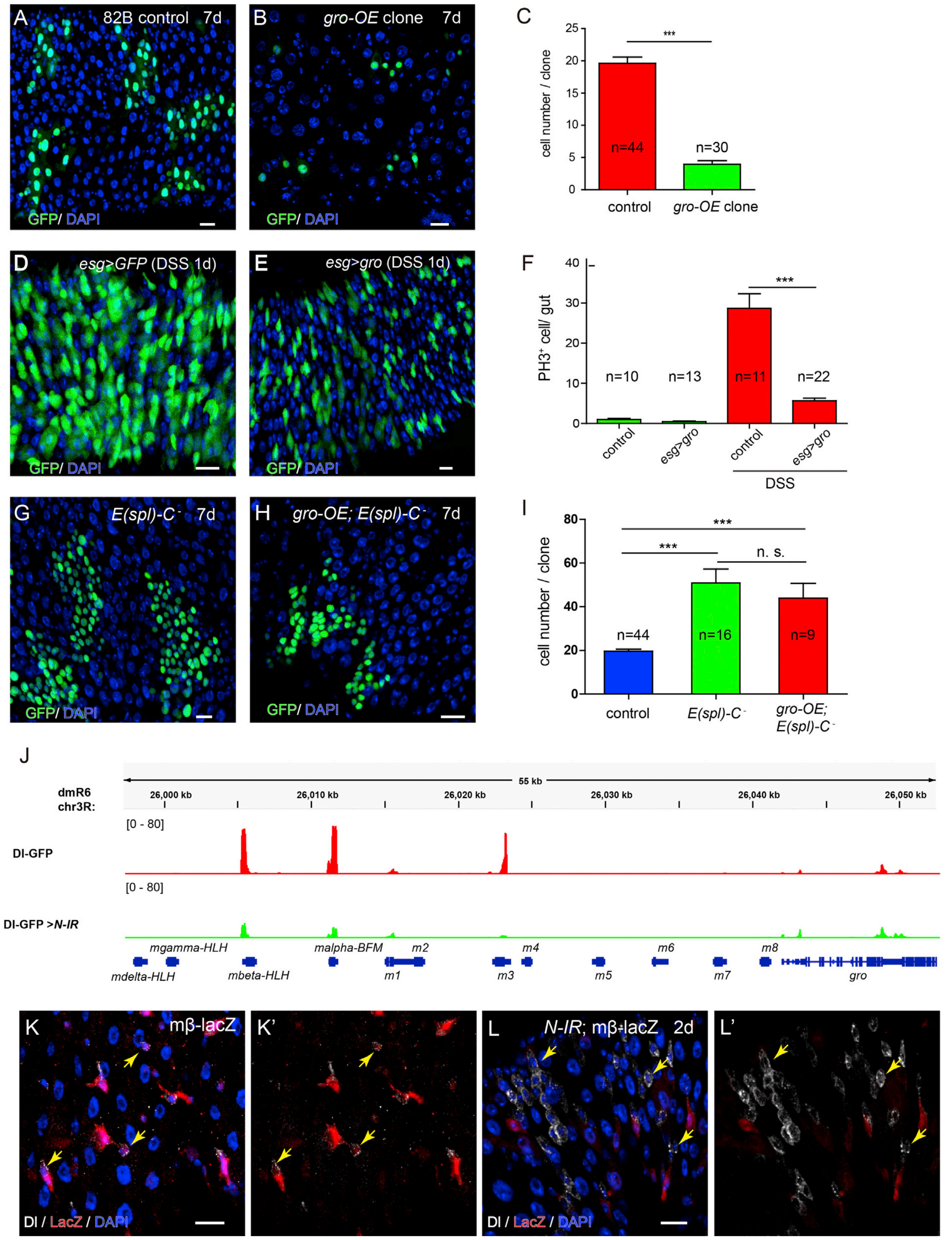

(legend on next page)

that individual *gro*-overexpressing clones were typically smaller (containing fewer cells) than the wild-type clones (Figures 6A–6C). Intestinal damage caused by DSS feeding effectively induces ISC proliferation and epithelial regeneration, as previously reported (Amcheslavsky et al., 2009). *Gro* overexpression was also able to significantly inhibit this damage-induced ISC proliferation (Figures 6D–6F). These observations suggest that *gro* is necessary and sufficient to inhibit ISC proliferation under both normal and stress conditions.

To test whether Gro also cooperates with E(spl)-C proteins to regulate ISC proliferation, we generated *P[gro⁺] Df*(3R) *gro32.2* mutant clones and found that *gro* overexpression failed to inhibit the growth of these *E(spl)-C* mutant clones (Figures 6G–6I). The results suggest that the proliferation-inhibitory effect of Gro requires E(spl)-C proteins, and therefore it is likely that Gro cooperates with E(spl)-C proteins to negatively regulate ISC proliferation.

If Gro indeed functions together with E(spl)-C proteins to limit ISC proliferation, these factors must be expressed in ISCs. To determine whether *E(spl)-C* genes are expressed in ISCs, and whether there is a baseline level of Notch activity in ISCs that may contribute to their expression, we compared the gene expression profile of ISCs with and without *Notch* depletion. Consistent with our previous observations (Chen et al., 2018), we found that several *E(spl)-C* genes, such as *mα*, *mβ*, and *m3*, were also expressed in ISCs (Figure 6J). In addition, the expression of *mα*, *mβ*, and *m3* were all significantly downregulated following *Notch* depletion (Figure 6J). This level of Notch target gene activation in ISCs is not detectable by NRE-lacZ, the typical Notch activation reporter that only marks EBs. Therefore, we further identified and examined a lacZ transcription reporter for *mβ*. Normally it was mainly expressed in EBs, and we found that it was also generally expressed in ISCs, albeit in a much lower level. Depletion of *Notch* completely abolished its expression in ISCs (Figures 6K and 6L).

To understand the functional requirement for *E(spl)-C* genes in ISCs, we knocked down *mα*, *mβ*, and *m3*, three most abundantly expressed *E(spl)-C* genes, in ISCs. As shown in Figure S7, individual knock down of either one of them did not cause any obvious phenotype (Figures S7B–S7D). However, simultaneous knocking down of *mα* and *mβ* or *mα* and *m3* caused moderate accumulation of progenitor cells (Figures S7E and S7F). Therefore, a baseline expression of E(spl)-C factors function redundantly in ISCs to restrict cell proliferation. Together with the observation that overexpressing mβ in ISCs failed to deplete Dl^+ cells in the absence of Gro (Figure S7G), these data collectively demonstrate that the E(spl)-C factors function cooperatively with a common co-repressor Gro to regulate ISC proliferation and differentiation.

We thus conclude that Gro has two severable functions respectively in ISCs and EBs. In ISCs, Gro cooperates with a low level of E(spl)-C proteins to limit ISC proliferation. In the immediate daughter EBs, abundant E(spl)-C proteins are induced by Notch activation via lateral inhibition, and Gro cooperates with these E(spl)-C proteins to repress Dl expression and prevent cell-cycle re-entry, thereby defining EB fate (Figure 7).

DISCUSSION

In this study, we have demonstrated that CtBP and Gro function separately in Dl-Notch-mediated cell fate decision between ISCs and EBs in the *Drosophila* ISC lineages. CtBP functions specifically in ISCs to participate in Su(H)-mediated transcriptional repression, whereas Gro functions specifically in EBs to participate in E(spl)-C-mediated transcriptional repression (Figure 7). As a result, loss of *CtBP* causes ISC differentiation and loss, whereas loss of *gro* causes ISC-like tumor development. Since both CtBP and Gro are highly conserved transcriptional co-repressors from flies to mammals, our findings may have important implications about the roles of these regulatory proteins in the lateral inhibition, stem cells, and tumorigenesis in mammals.

This distinctive division of labor for CtBP and Gro in lateral inhibition is interesting, because these two

Figure 6. *Gro* Mediates a Baseline Notch Activity in ISCs to Inhibit Cell Proliferation
(A and B) Compared with wild-type clones (A), *gro* overexpression clones grow much smaller in size (B).
(C) Quantification of cell numbers in wild-type and *gro*-overexpressed clones.
(D and E) DSS injury induces repaid proliferation in the intestine (D), while *Gro* overexpression inhibits DSS-induced ISC proliferation (E).
(F) Quantification of $PH3^+$ cells in control and *gro*-overexpressed guts after DSS treatment.
(G and H) *Gro* overexpression (H) fails to repress the growth of tumorous clones caused by the removal of *E(spl)-C* genes (G).
(I) Quantification of cell numbers in *E(spl)* mutant clones with or without *gro* overexpression.
(J) Comparative analysis of *E(spl)-C* gene expression in control (red) and *Notch-IR* (green) guts.
(K–L) Expression of mβ-lacZ could be detected in normal Dl^+ ISCs (yellow arrows) (K), but not in *Notch*-depleted Dl^+ ISCs (yellow arrows) (L).
Error bars indicate mean ± SEM. n.s., not significant, ***p < 0.001 (Student's t test). Scale bars, 20 μm.

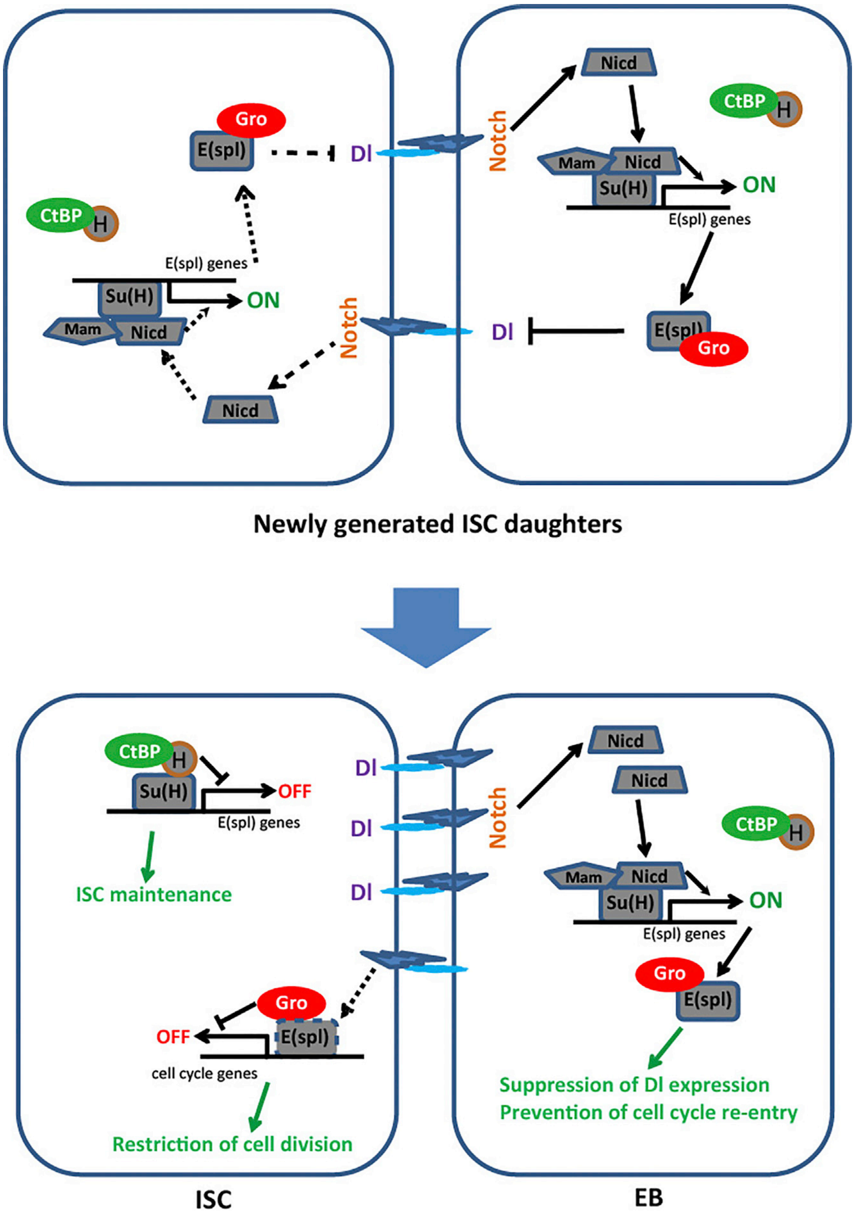

Figure 7. A Model for the Functions of CtBP and Gro in Lateral Inhibition between ISC and EB

After each ISC division, the fate of the two newly formed ISC daughters is specified by Notch-mediated lateral inhibition. Through lateral inhibition, a slight difference in Dl-Notch-Su(H)-E(spl)-C signaling cascade between the two identical daughters leads to two distinct cell fates. The daughter that retains Dl expression will remain as a new ISC, whereas the daughter with Notch activated will become an EB. Two co-repressors CtBP and Gro are both expressed generally in ISCs and EBs, but they have distinct functions in the process of lateral inhibition. CtBP specifically participates in Su(H) and Hairless-mediated transcriptional repression of Notch targets in ISCs, thereby maintaining ISC fate. Gro has two separate roles respectively in ISCs and EBs. In EBs, it is in complex with E(spl)-C factors to suppress Dl expression and prevent cell-cycle re-entry. In ISCs, this protein also cooperates with E(spl)-C factors (that are expressed at low levels in ISCs) to restrict ISC proliferation.

global co-repressors are generally believed to function simultaneously as negative regulators of Notch signaling by mediating Su(H)-mediated transcriptional repression. Biochemically, CtBP and Gro are able to simultaneously interact with Su(H) through separate binding motifs. Genetically, removing one functional copy of *gro* or *CtBP* function enhances the bristle loss phenotype caused by *hairless* mutation (Barolo et al., 2002). However, because Gro also interacts and cooperates with E(spl)-C proteins, and therefore Gro is also considered as a positive regulator of Notch signaling (Paroush et al., 1994). This dual function of Gro could make the genetic phenotypes too complicated to accurately interpret, not to mention their pleotropic functions as they bind to many other transcription factors and mediate transcriptional output of many additional signaling pathways (Apidianakis et al., 2001; Cavallo et al., 1998; Hanson et al., 2012; Hasson et al., 2005). We thus argue that reliable characterization of Gro and CtBP in any lateral inhibition processes requires cell-type-specific examination of gene function, in addition to the genetic interaction experiments.

In the *Drosophila* midgut, it has long been observed that each individual ISC is able to divide either symmetrically (to produce two ISCs or two EBs) or asymmetrically (to produce one ISC and one EB). At the anaphase of mitosis, Dl is found to be symmetrically segregated into two

daughters, but in the case of asymmetric division, Dl is specifically downregulated in one of the daughter cells, to produce an asymmetric outcome (Ohlstein and Spradling, 2007). Recent analysis further supports this idea by showing that ISCs commonly divide symmetrically, but whether it is a symmetric or asymmetric outcome depends on cell competition between the two daughters via Dl-Notch-mediated lateral inhibition (de Navascues et al., 2012). As several canonical Notch signaling components are involved in ISC-EB separation (Bardin et al., 2010; Ohlstein and Spradling, 2007), the requirements for CtBP and Gro in the process reported here further support the idea that Dl-Notch-mediated lateral inhibition separates ISC and EB fate. It has been shown that Hairless is specifically required in ISCs to maintain their fate, a function similar to CtBP reported here (Bardin et al., 2010). This is consistent with the idea that Hairless acts an adaptor to link Su(H) and CtBP to establish the repressor complex (Barolo et al., 2002). In addition to ISC and EB fate separation, Gro is also essential for EE generation. Loss of Gro in ISCs causes formation of many ISC-like cell clusters, and these cells are able to differentiate further into ECs, but not EEs. This phenotype is largely reminiscent of the phenotype caused by the loss of *E(spl)-C* genes. On one hand, similar phenotypes support a role for Gro as an indispensable co-repressor for E(spl)-C proteins. On the other hand, the EE-less phenotype dos not seem to support a role for E(spl)-C in favoring EC fate by opposing EE fate. The underlying mechanism remains to be investigated, but one potential explanation is that there are additional Notch targets, in addition to *E(spl)-C* genes, which function to promote EC differentiation, and that these additional Notch targets could be compensatorily upregulated on the loss of *E(spl)-C* genes. This may lead to the priming of ISCs to EC fate instead of EE fate. Consistent with this idea, the *AS-C* gene *sc*, which is essential for EE specification, is upregulated in ISCs when Notch is depleted, but is not when *E(spl)-C* genes are depleted (Chen et al., 2018).

This study also reveals a baseline level of Notch activity in ISCs that contribute to the expression of E(spl)-C factors, which cooperates with Gro to limit ISC proliferation. Previous studies suggest that Notch is mainly activated in EBs to promote cell differentiation, but subsequent RNA-seq analysis revealed that many *E(spl)-C* genes are expressed in both ISCs and EBs (Dutta et al., 2015). Furthermore, Notch is believed to be activated in ISCs at a particular time window when EE is generated, and this Notch activity induced by a Dl ligand from the newly formed EE is important for maintaining ISC multipotency (Guo and Ohlstein, 2015). By comparing RNA-seq data of normal ISCs and *Notch*-depleted ISCs combined with reporter analysis, here we show that, although the expression of *E(spl)-C* genes in ISCs is attributed to Notch activity in ISCs, these Notch-dependent *E(spl)-C* genes seem to be generally expressed at a baseline level in all ISCs. By contrast, we have recently shown that *E(spl)m8*, as well as several other *E(spl)-C* genes, are oscillatorily expressed in a regulatory feedback loop with *AS-C* genes that controls the fate of ISC daughters (Chen et al., 2018). We propose that Notch-independent expression of *E(spl)-C* genes in ISCs serves in a feedback loop to control the binary fate decision of ISCs, while Notch-dependent expression of E(spl)-C proteins, which is at a low level, function cooperatively with Gro to suppress the expression of cell-cycle genes, such as *string* and *cyclin B*, thereby limiting ISC activity at a baseline level. This function of Gro and E(spl)-C could provide additional levels of regulation on ISC activity in response to environmental changes. It has been shown that Gro is phosphorylated on epidermal growth factor receptor (EGFR) activation, and the phosphorylation on Gro attenuates Gro-E(spl) complex-mediated transcriptional silencing (Hasson et al., 2005). EGFR/mitogen-activated protein kinase (MAPK) signaling is one of the major pathways that promotes ISC proliferation and epithelial regeneration in the *Drosophila* midgut (Biteau and Jasper, 2011; Buchon et al., 2010; Jiang et al., 2011; Liang et al., 2017; Xu et al., 2011). In addition, increased EGFR pathway activity is also necessary for Notch loss-induced ISC-like tumor growth (Patel et al., 2015). It is thus possible that Gro could also possibly participate in EGFR/MAPK activity-induced ISC proliferation to regulate homeostatic and regenerative epithelial turnover.

EXPERIMENTAL PROCEDURES

Fly Strains and Cultivation

Fly stocks were cultivated on standard food and kept at 25°C unless otherwise stated. The following fly strains were used: *UAS-gro-RNAi* no. 1 (VDRC, v110546/KK108953); *UAS-gro-RNAi* no. 2 (Tsinghua Fly Center, no. 1717/HMS01506); UAS-*stg-RNAi* (BDSC, no. 34831); *UAS-CtBP-RNAi* (Tsinghua Fly Center, no. 1919/HMS00677); UAS-*Notch-RNAi* (BDSC, no. 7078); *UAS-ttk-RNAi* (VDRC, v10855); *UAS-m3-RNAi* (BDSC, no. 34831); *UAS-mα-RNAi* (Bloomington *Drosophila* Stock Center [BDSC], no. 34831); *UAS-mβ-RNAi* (BDSC, no. 34831); *gro*^{E48} (a gift from Barbara Jennings); *CtBP*$^{87De-10}$ (BDSC, no. 1663); *sna*^{18} (BDSC, no. 2311); *esg*$^{35CE-1}$(BDSC, no. 3900); *NRE-lacZ* (Gbe-Su(H)m8-lacZ, a gift from Sarah Bray)*; P[gro+]Df*(3R) *gro*$^{32.2}$ (BDSC, no. 52011); *mβ-LacZ* (a gift from Renjie Jiao); *Dl-Gal4* (an ISC-specific driver)*; NRE-Gal4* (an EB-specific driver, a gift from Steven Hou) (Zeng et al., 2010); *esg-Gal4, UAS-GFP* (a gift from Shigeo Hayashi); *UAS-Nicd* (a gift from Ting Xie); *UAS-Hairless* (BDSC, no. 15672); *UAS-m7* (BDSC, no. 26681); *UAS-mβ* (BDSC, no. 26675); *UAS-sc* (BDSC, no. 26687).

Generation of *UAS-gro-FLAG* transgenic flies: the *gro* cDNA was cloned into the attB-pUAST-3*Flag vector, sequence verified, and

subsequently inserted into attP40 or attP2 sites of phiC31 stocks via standard microinjection. Both insertion lines produced a similar effect in this study.

Generation of *UAS-Hairless*$^{\Delta C}$ transgenic flies: primers were used as described previously (Barolo et al., 2002). The truncated ORF of *Hairless* were amplified and cloned into attB-pUAST vector. Microinjection was then carried out and inserted this element into the attP2 site.

Mosaic Analysis

Cell-type-specific gene depletion or overexpression was conducted using the Gal4/UAS/Gal80 system (Brand and Perrimon, 1993; McGuire et al., 2004). Crosses were carried out at 18°C, and 3- to 5-day-old progenies with the correct genotypes were transferred to 29°C for 7 days before dissection and staining, unless otherwise noted.

Cell clones were generated using the MARCM system (Lee and Luo, 1999), and the flp-out technique (Struhl and Basler, 1993) was used for cell lineage-tracing experiments, as described previously (Lin et al., 2008; Wang et al., 2015; Xu et al., 2011).

Immunostaining

Immunostaining of *Drosophila* midgut was performed as described previously (Lin et al., 2008). In brief, 10–15 female midguts were dissected in PBS and fixed in 4% paraformaldehyde for 30 min at room temperature. After dehydration in methanol and rehydrated in PBT containing 0.1% Triton X-100, primary antibodies were added in 5% NGS-PBT solution and incubated overnight at 4°C. Secondary antibodies were incubated at room temperature for 2 h, followed by 5 min DAPI staining. The intestines were mounted using 70% glycerol and the images captured with a Nikon AR1 confocal microscope. Images were adjusted in Adobe Photoshop and assembled in Adobe Illustrator. All scale bars indicate 20 μm unless otherwise noted.

Primary antibodies listed below were used in this study: mouse anti-Dl (Development Studies Hybridoma Bank [DSHB]; 1:300); mouse anti-Pros (DSHB; 1:300); mouse/rabbit anti-phospho-histone H3 (Cell Signaling Technology, no. 9706, 1:1,000); rabbit polyclonal anti-β-galactosidase (Cappel, 0855976; 1:6,000); rabbit anti-Pdm1 (a gift from Xiaohang Yang, Zhejiang University, China; 1:1,000). Secondary antibodies include goat anti-rabbit, anti-mouse IgGs conjugated with Alexa Fluor 488, Alexa Fluor 568, or Cy5 (Molecular Probes, A11034-A11036, A10524; 1:300).

RNA-Seq

Dl-Gal4, UAS-GFP; Gal80^{ts} stock was used to specifically express *gro-RNAi* (experimental group) or GFP (control group) in ISCs. Crosses were performed at 18°C, and 3- to 5-day-old F1 progenies with the correct genotypes were transferred to 29°C for 4 days before dissection. For each replicate, approximately 200 midguts were dissected and three replicates were collected for each genotype. Tissue digestion, cell sorting, and RNA isolation were carried out according to previously described protocol (Chen et al., 2016; Dutta et al., 2013). For each sample, about 30,000 PI^{-}, GFP^{+} cells were isolated and collected in a 1.5-mL tube containing 400 μL RNA extraction buffer. An Arcturus PicoPure RNA isolation kit (Applied Biosystems) and an Arcturus RiboAmp HS PLUS RNA amplification kit were used for RNA extraction and amplification separately. Libraries were then generated for deep-sequencing on the Illumina GA II instrument.

qRT-PCR

For each sample, 1 μg of amplified RNA described above was used to synthesize cDNA using 5× All-In-One RT MasterMix (ABM, cat. no. G485). qRT-PCR was then carried out using the SYBR PrimeScript RT-PCR Kit (Takara) on an ABI PRISM 7500 Fast Real-Time PCR System (Applied Biosystems). Expression levels of selected genes were normalized to *gapdh*, and RT-PCR primers used in this study as following:

E(spl)-C genes
- m3_F GCCTGATGATTGCGGTATTT
- m3_R ATGCTGCCGATCAGATTACC
- m5_F TGGTTTCTTCGACTGGCTTG
- m5_R CCAGACTTCTGTTACAACCTCCA
- m7_F GACTGATGGAGGAGCAGGAG
- m7_R GTGGCTTTTGGAACCACACT
- m8_F GCTGTGATATCCGGAGGAG
- m8_R AATTCCACGAAGCACAGTCC
- mb_F ACCAAGATGGAGGACGACAG
- mb_R CAGCCAGCAGAAAAGGAAGT
- mr_F CACTCCACCACCCTCTGAAT
- mr_R CATCGTCTCAACTACCTGCAA
- md_F CTCTTCTCGCGGAGACTTTG
- md_R CACCAGCTCAAGGACATGAA
- m2_F TCAATGAGCAACTCCTGCTG
- m2_R CATGCGTAACGTGTGGAAAC
- m4_F GTCCTCAATTTCGCAGGACT
- m4_R GGAGCAGAACCTCAAGAACG
- ma_F GTTCGAGATTGTCGAGGAGC
- ma_R CCAGCTACAGCATCAAGCAG

stg:
- stg_F ACACTCGCATTCATGCAAAACA
- stg_R AGCGTAAATTGTACCTAGCAGA

Dl:
- Dl_F CATTTGCTTCACAGTCATCGTG
- Dl_R GCTTTAGGCAGACGCGAAAC

gro:
- gro-F GGCTGTGGGTATGGAGAACT
- gro-R TCGTGCAGATGCAGTTGATA

GAPDH:
- GAPDH_F GTCGGGCTTGTAGGCATCC
- GAPDH_R AGGCATCCACTCACTTGAAGG

Statistical Analysis

All data were presented in the form of mean ± SEM. GraphPad Prism 5 software (GraphPad Software) was used to calculate p values by unpaired Student's t test.

ACCESSION NUMBERS

The accession number for the RNA-sequencing data of Dl-GFP and *gro-RNAi* and *Notch-RNAi* reported in this paper is GEO: GSE112331.

SUPPLEMENTAL INFORMATION

Supplemental Information can be found online at https://doi.org/10.1016/j.stemcr.2019.03.005.

AUTHOR CONTRIBUTIONS

X.G. and R.X. conceived and designed the experiments, analyzed the data, and wrote the manuscript. X.G. and Z.Y. performed the experiments. H.H. and T.C. performed the bioinformatics analysis.

ACKNOWLEDGMENTS

We thank the members of the fly community, as cited in the Materials and Methods, for generously providing fly strains and antibodies; the Bloomington *Drosophila* Stock Center (BDSC), the Tsinghua Fly Center, the Vienna *Drosophila* RNAi Center (VDRC) and Development Studies Hybridoma Bank (DSHB) for reagents; John Snyder for editing the manuscript, and the Xi lab members for comments and discussions. This work was supported by a National Key Research and Development Program of China (2017YFA0103602) and a National Basic Research Program of China (2014CB850002) from the Chinese Ministry of Science and Technology.

Received: April 1, 2018
Revised: March 15, 2019
Accepted: March 15, 2019
Published: April 11, 2019

REFERENCES

Amcheslavsky, A., Jiang, J., and Ip, Y.T. (2009). Tissue damage-induced intestinal stem cell division in *Drosophila*. Cell Stem Cell *4*, 49–61.

Apidianakis, Y., Grbavec, D., Stifani, S., and Delidakis, C. (2001). Groucho mediates a Ci-independent mechanism of hedgehog repression in the anterior wing pouch. Development *128*, 4361–4370.

Artavanis-Tsakonas, S., Rand, M.D., and Lake, R.J. (1999). Notch signaling: cell fate control and signal integration in development. Science *284*, 770–776.

Bardin, A.J., Perdigoto, C.N., Southall, T.D., Brand, A.H., and Schweisguth, F. (2010). Transcriptional control of stem cell maintenance in the *Drosophila* intestine. Development *137*, 705–714.

Barolo, S., Stone, T., Bang, A.G., and Posakony, J.W. (2002). Default repression and Notch signaling: hairless acts as an adaptor to recruit the corepressors Groucho and dCtBP to Suppressor of Hairless. Genes Dev. *16*, 1964–1976.

Biteau, B., and Jasper, H. (2011). EGF signaling regulates the proliferation of intestinal stem cells in *Drosophila*. Development *138*, 1045–1055.

Biteau, B., and Jasper, H. (2014). Slit/Robo signaling regulates cell fate decisions in the intestinal stem cell lineage of *Drosophila*. Cell Rep. *7*, 1867–1875.

Brand, A.H., and Perrimon, N. (1993). Targeted gene expression as a means of altering cell fates and generating dominant phenotypes. Development *118*, 401–415.

Bray, S.J. (2006). Notch signalling: a simple pathway becomes complex. Nat. Rev. Mol. Cell Biol. *7*, 678–689.

Buchon, N., Broderick, N.A., Kuraishi, T., and Lemaitre, B. (2010). *Drosophila* EGFR pathway coordinates stem cell proliferation and gut remodeling following infection. BMC Biol. *8*, 152.

Cavallo, R.A., Cox, R.T., Moline, M.M., Roose, J., Polevoy, G.A., Clevers, H., Peifer, M., and Bejsovec, A. (1998). Drosophila Tcf and Groucho interact to repress Wingless signalling activity. Nature *395*, 604–608.

Chanet, S., and Schweisguth, F. (2012). Regulation of epithelial polarity by the E3 ubiquitin ligase neuralized and the Bearded inhibitors in Drosophila. Nat. Cell Biol. *14*, 467–476.

Chen, J., Xu, N., Huang, H., Cai, T., and Xi, R. (2016). A feedback amplification loop between stem cells and their progeny promotes tissue regeneration and tumorigenesis. Elife *5*. https://doi.org/10.7554/eLife.14330.

Chen, J., Xu, N., Wang, C., Huang, P., Huang, H., Jin, Z., Yu, Z., Cai, T., Jiao, R., and Xi, R. (2018). Transient Scute activation via a self-stimulatory loop directs enteroendocrine cell pair specification from self-renewing intestinal stem cells. Nat. Cell Biol. *20*, 152–161.

Courey, A.J., and Jia, S. (2001). Transcriptional repression: the long and the short of it. Genes Dev. *15*, 2786–2796.

de Navascues, J., Perdigoto, C.N., Bian, Y., Schneider, M.H., Bardin, A.J., Martinez-Arias, A., and Simons, B.D. (2012). *Drosophila* midgut homeostasis involves neutral competition between symmetrically dividing intestinal stem cells. EMBO J. *31*, 2473–2485.

Dietzl, G., Chen, D., Schnorrer, F., Su, K.C., Barinova, Y., Fellner, M., Gasser, B., Kinsey, K., Oppel, S., Scheiblauer, S., et al. (2007). A genome-wide transgenic RNAi library for conditional gene inactivation in *Drosophila*. Nature *448*, 151–156.

Dutta, D., Dobson, A.J., Houtz, P.L., Glasser, C., Revah, J., Korzelius, J., Patel, P.H., Edgar, B.A., and Buchon, N. (2015). Regional cell-specific transcriptome mapping reveals regulatory complexity in the adult *Drosophila* midgut. Cell Rep. *12*, 346–358.

Dutta, D., Xiang, J., and Edgar, B.A. (2013). RNA expression profiling from FACS-isolated cells of the *Drosophila* intestine. Curr. Protoc. Stem Cell Biol. *27*, Unit 2F.2.

Furriols, M., and Bray, S. (2001). A model Notch response element detects Suppressor of Hairless-dependent molecular switch. Curr. Biol. *11*, 60–64.

Gierer, A., and Meinhardt, H. (1972). A theory of biological pattern formation. Kybernetik *12*, 30–39.

Guo, Z., and Ohlstein, B. (2015). Stem cell regulation. Bidirectional Notch signaling regulates *Drosophila* intestinal stem cell multipotency. Science *350*. https://doi.org/10.1126/science.aab0988.

Hanson, A.J., Wallace, H.A., Freeman, T.J., Beauchamp, R.D., Lee, L.A., and Lee, E. (2012). XIAP monoubiquitylates Groucho/TLE to promote canonical Wnt signaling. Mol. Cell *45*, 619–628.

Hasson, P., Egoz, N., Winkler, C., Volohonsky, G., Jia, S., Dinur, T., Volk, T., Courey, A.J., and Paroush, Z. (2005). EGFR signaling attenuates Groucho-dependent repression to antagonize Notch transcriptional output. Nat. Genet. *37*, 101–105.

Jennings, B.H., Pickles, L.M., Wainwright, S.M., Roe, S.M., Pearl, L.H., and Ish-Horowicz, D. (2006). Molecular recognition of transcriptional repressor motifs by the WD domain of the Groucho/TLE corepressor. Mol. Cell *22*, 645–655.

Jiang, H., Grenley, M.O., Bravo, M.J., Blumhagen, R.Z., and Edgar, B.A. (2011). EGFR/Ras/MAPK signaling mediates adult midgut epithelial homeostasis and regeneration in *Drosophila*. Cell Stem Cell *8*, 84–95.

Kopan, R., and Ilagan, M.X. (2009). The canonical Notch signaling pathway: unfolding the activation mechanism. Cell *137*, 216–233.

Korzelius, J., Naumann, S.K., Loza-Coll, M.A., Chan, J.S., Dutta, D., Oberheim, J., Glasser, C., Southall, T.D., Brand, A.H., Jones, D.L., et al. (2014). Escargot maintains stemness and suppresses differentiation in *Drosophila* intestinal stem cells. EMBO J. *33*, 2967–2982.

Lai, E.C. (2004). Notch signaling: control of cell communication and cell fate. Development *131*, 965–973.

Lai, E.C., Deblandre, G.A., Kintner, C., and Rubin, G.M. (2001). *Drosophila* neuralized is a ubiquitin ligase that promotes the internalization and degradation of delta. Dev. Cell *1*, 783–794.

Lecourtois, M., and Schweisguth, F. (1995). The neurogenic suppressor of hairless DNA-binding protein mediates the transcriptional activation of the enhancer of split complex genes triggered by Notch signaling. Genes Dev. *9*, 2598–2608.

Lee, T., and Luo, L. (1999). Mosaic analysis with a repressible cell marker for studies of gene function in neuronal morphogenesis. Neuron *22*, 451–461.

Li, Y., Pang, Z., Huang, H., Wang, C., Cai, T., and Xi, R. (2017). Transcription factor antagonism controls enteroendocrine cell specification from intestinal stem cells. Sci. Rep. *7*, 988.

Liang, J., Balachandra, S., Ngo, S., and O'Brien, L.E. (2017). Feedback regulation of steady-state epithelial turnover and organ size. Nature *548*, 588–591.

Lin, G., Xu, N., and Xi, R. (2008). Paracrine Wingless signalling controls self-renewal of *Drosophila* intestinal stem cells. Nature *455*, 1119–1123.

Loza-Coll, M.A., Southall, T.D., Sandall, S.L., Brand, A.H., and Jones, D.L. (2014). Regulation of *Drosophila* intestinal stem cell maintenance and differentiation by the transcription factor Escargot. EMBO J. *33*, 2983–2996.

McGuire, S.E., Mao, Z., and Davis, R.L. (2004). Spatiotemporal gene expression targeting with the TARGET and gene-switch systems in *Drosophila*. Sci. STKE *2004*. https://doi.org/10.1126/stke.2202004pl6.

Micchelli, C.A., and Perrimon, N. (2006). Evidence that stem cells reside in the adult *Drosophila* midgut epithelium. Nature *439*, 475–479.

Morel, V., Lecourtois, M., Massiani, O., Maier, D., Preiss, A., and Schweisguth, F. (2001). Transcriptional repression by suppressor of hairless involves the binding of a hairless-dCtBP complex in *Drosophila*. Curr. Biol. *11*, 789–792.

Nagel, A.C., Krejci, A., Tenin, G., Bravo-Patiño, A., Bray, S., Maier, D., and Preiss, A. (2005). Hairless-mediated repression of Notch target genes requires the combined activity of Groucho and CtBP corepressors. Mol. Cell. Biol. *25*, 10433–10441.

Nagel, A.C., and Preiss, A. (2011). Fine tuning of Notch signaling by differential co-repressor recruitment during eye development of *Drosophila*. Hereditas *148*, 77–84.

Nagel, A.C., Wech, I., Schwinkendorf, D., and Preiss, A. (2007). Involvement of co-repressors Groucho and CtBP in the regulation of single-minded in *Drosophila*. Hereditas *144*, 195–205.

Ni, J.Q., Zhou, R., Czech, B., Liu, L.P., Holderbaum, L., Yang-Zhou, D., Shim, H.S., Tao, R., Handler, D., Karpowicz, P., et al. (2011). A genome-scale shRNA resource for transgenic RNAi in *Drosophila*. Nat. Methods *8*, 405–407.

Nibu, Y., Zhang, H., Bajor, E., Barolo, S., Small, S., and Levine, M. (1998). dCtBP mediates transcriptional repression by Knirps, Kruppel and Snail in the *Drosophila* embryo. EMBO J. *17*, 7009–7020.

Ohlstein, B., and Spradling, A. (2006). The adult *Drosophila* posterior midgut is maintained by pluripotent stem cells. Nature *439*, 470–474.

Ohlstein, B., and Spradling, A. (2007). Multipotent *Drosophila* intestinal stem cells specify daughter cell fates by differential notch signaling. Science *315*, 988–992.

Paroush, Z., Finley, R.L., Jr., Kidd, T., Wainwright, S.M., Ingham, P.W., Brent, R., and Ish-Horowicz, D. (1994). Groucho is required for *Drosophila* neurogenesis, segmentation, and sex determination and interacts directly with hairy-related bHLH proteins. Cell *79*, 805–815.

Patel, P.H., Dutta, D., and Edgar, B.A. (2015). Niche appropriation by *Drosophila* intestinal stem cell tumours. Nat. Cell Biol. *17*, 1182–1192.

Pavlopoulos, E., Pitsouli, C., Klueg, K.M., Muskavitch, M.A., Moschonas, N.K., and Delidakis, C. (2001). Neuralized encodes a peripheral membrane protein involved in delta signaling and endocytosis. Dev. Cell *1*, 807–816.

Poortinga, G., Watanabe, M., and Parkhurst, S.M. (1998). *Drosophila* CtBP: a Hairy-interacting protein required for embryonic segmentation and hairy-mediated transcriptional repression. EMBO J. *17*, 2067–2078.

Sancho, R., Cremona, C.A., and Behrens, A. (2015). Stem cell and progenitor fate in the mammalian intestine: Notch and lateral inhibition in homeostasis and disease. EMBO Rep. *16*, 571–581.

Simpson, P. (1990). Lateral inhibition and the development of the sensory bristles of the adult peripheral nervous system of *Drosophila*. Development *109*, 509–519.

Struhl, G., and Basler, K. (1993). Organizing activity of wingless protein in *Drosophila*. Cell *72*, 527–540.

Voog, J., Sandall, S.L., Hime, G.R., Resende, L.P., Loza-Coll, M., Aslanian, A., Yates, J.R., 3rd, Hunter, T., Fuller, M.T., and Jones, D.L. (2014). Escargot restricts niche cell to stem cell conversion in the *Drosophila* testis. Cell Rep. *7*, 722–734.

Wakamatsu, Y., Maynard, T.M., and Weston, J.A. (2000). Fate determination of neural crest cells by NOTCH-mediated lateral inhibition and asymmetrical cell division during gangliogenesis. Development *127*, 2811.

Wang, C., Guo, X., Dou, K., Chen, H., and Xi, R. (2015). Ttk69 acts as a master repressor of enteroendocrine cell specification in

Drosophila intestinal stem cell lineages. Development *142*, 3321–3331.

Wu, L., Aster, J.C., Blacklow, S.C., Lake, R., Artavanis-Tsakonas, S., and Griffin, J.D. (2000). MAML1, a human homologue of *Drosophila* mastermind, is a transcriptional co-activator for NOTCH receptors. Nat. Genet. *26*, 484–489.

Xu, N., Wang, S.Q., Tan, D., Gao, Y., Lin, G., and Xi, R. (2011). EGFR, Wingless and JAK/STAT signaling cooperatively maintain *Drosophila* intestinal stem cells. Dev. Biol. *354*, 31–43.

Yin, C., and Xi, R. (2018). A phyllopod-mediated feedback loop promotes intestinal stem cell enteroendocrine commitment in *Drosophila*. Stem Cell Reports *10*, 43–57.

Zeng, X., Chauhan, C., and Hou, S.X. (2010). Characterization of midgut stem cell- and enteroblast-specific Gal4 lines in *Drosophila*. Genesis *48*, 607–611.

Zeng, X., and Hou, S.X. (2015). Enteroendocrine cells are generated from stem cells through a distinct progenitor in the adult *Drosophila* posterior midgut. Development *142*, 644–653.

Stem Cell Reports, Volume *12*

Supplemental Information

Division of Labor: Roles of Groucho and CtBP in Notch-Mediated Lateral Inhibition that Controls Intestinal Stem Cell Differentiation in *Drosophila*

Xingting Guo, Huanwei Huang, Ziqing Yang, Tao Cai, and Rongwen Xi

Supplemental Data

Division of labor: roles of Groucho and CtBP in Notch-mediated lateral inhibition that controls intestinal stem cell differentiation in *Drosophila*

Xingting Guo[1, 2], Huanwei Huang[2], Ziqing Yang[2], Tao Cai[2] and Rongwen Xi[2, 3 *]

[1] College of Life Sciences, Beijing Normal University, Beijing 100875, China;

[2] National Institute of Biological Sciences, No. 7 Science Park Road, Zhongguancun Life Science Park, Beijing 102206, China

[3] Tsinghua Institute of Multidisciplinary Biomedical Research, Tsinghua University, Beijing, China.

* Correspondence: *xirongwen@nibs.ac.cn*

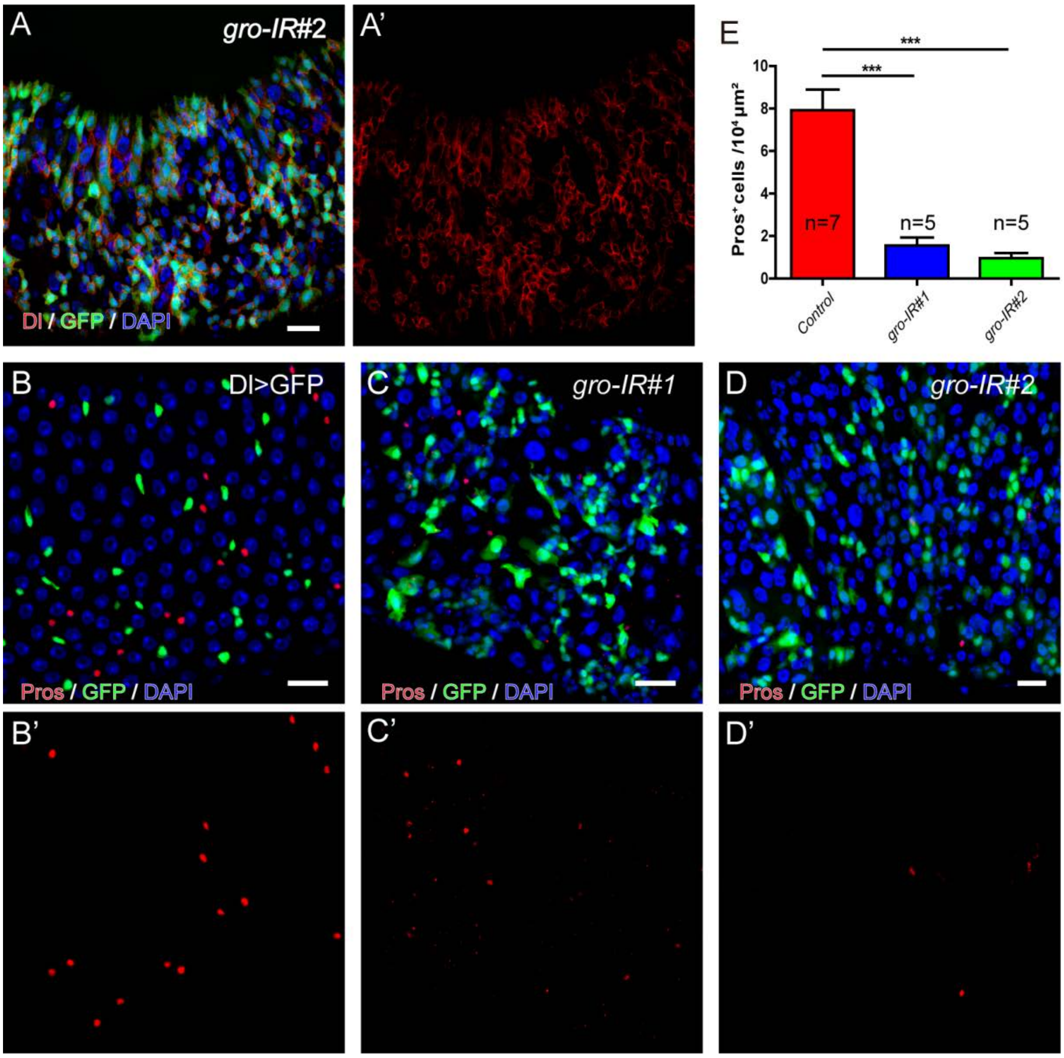

Figure S1, Related to Figure 1. Accumulation of Dl$^+$ cells and reduced EE generation following the depletion of *gro* in ISCs.

(A) Knocking down *gro* in ISCs using another RNAi line (#HMS01506) leads to similar accumulation of Dl$^+$ cells. **(B-D)** Compared to control guts, knocking down *gro* in ISCs using two independent RNAi lines causes significant reduction of Pros$^+$ cells in the intestinal epithelium. **(E)** Quantification of the density of Pros$^+$ cells in posterior midgut. Error bars indicate mean ± s.e.m. and the number of intestines calculated is labeled on the columns. ***P<0.001 (Student's t-test). Scale bars: 20

μm.

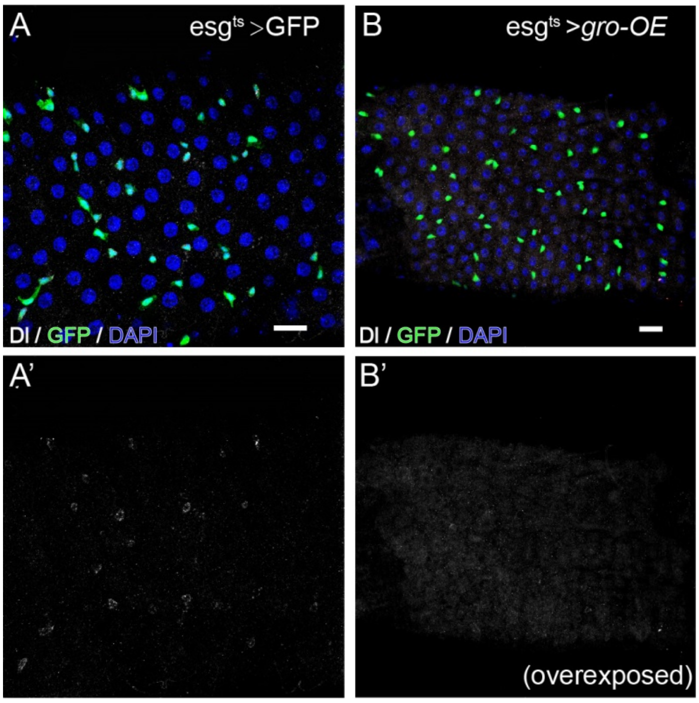

Figure S2, Related to Figure 1. *Gro* overexpression down-regulates Dl expression. Compared to control **(A)**, Ectopic expression of *gro* in esg^{+} cells (GFP, green) rapidly down-regulates Dl expression to an undetectable level **(B)**. Note that image overexposure causes increased background signal in **B'**.

-

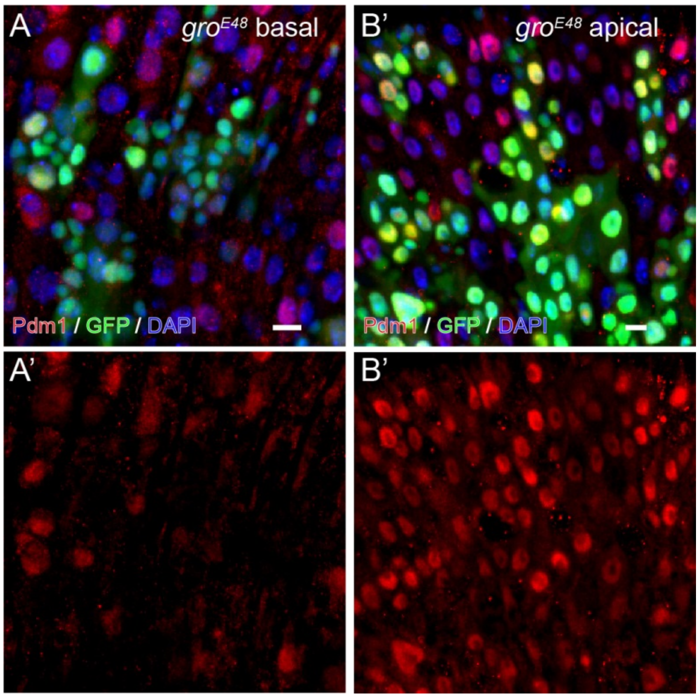

Figure S3, Related to Figure 1. The organization of Pdm1$^+$ EC cells in *gro^{E48}* mutant clones.

(A-B) Pdm1$^+$ polyploid cells mainly localized at apical layer (B) in the mutant clones, while most cells at basal layer (A) are Pdm1$^-$ diploid cells.

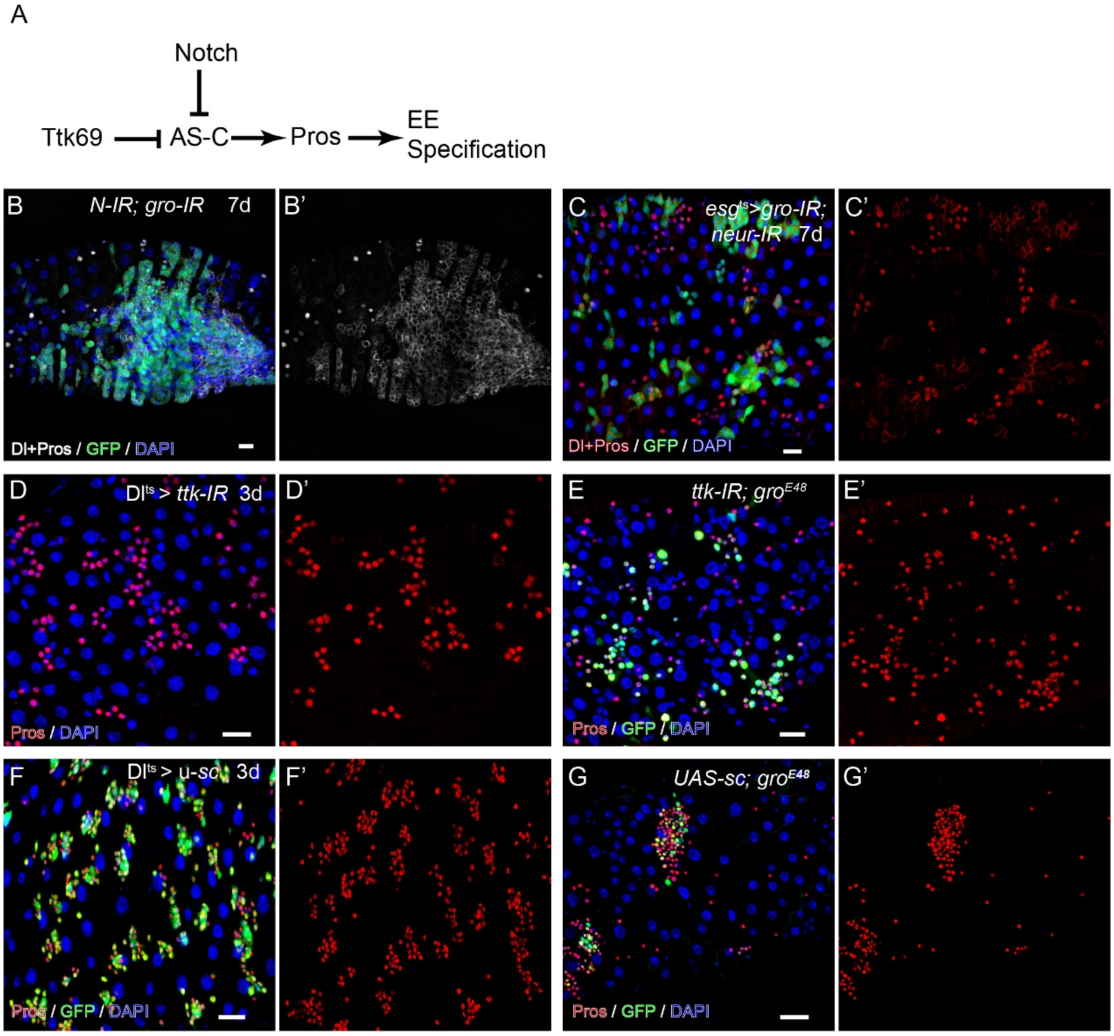

Figure S4, Related to Figure 1. *Gro* functions downstream of Notch and upstream of *ttk* and *sc* in regulating EE differentiation.

(A) A diagram of known regulatory pathways that control EE specification. **(B)** *Gro* depletion suppresses supernumerary EE generation in *Notch-RNAi* epithelium. **(C)** Loss of *gro* could not repress *neur-IR* induced supernumerary EE generation. **(D-G)** Either knocking down *ttk* or over-expressing *sc* is sufficient to cause supernumerary EE generation from ISCs, and depletion of *gro* does not prevent supernumerary EE phenotype caused by either *ttk* depletion or *sc* overexpression.

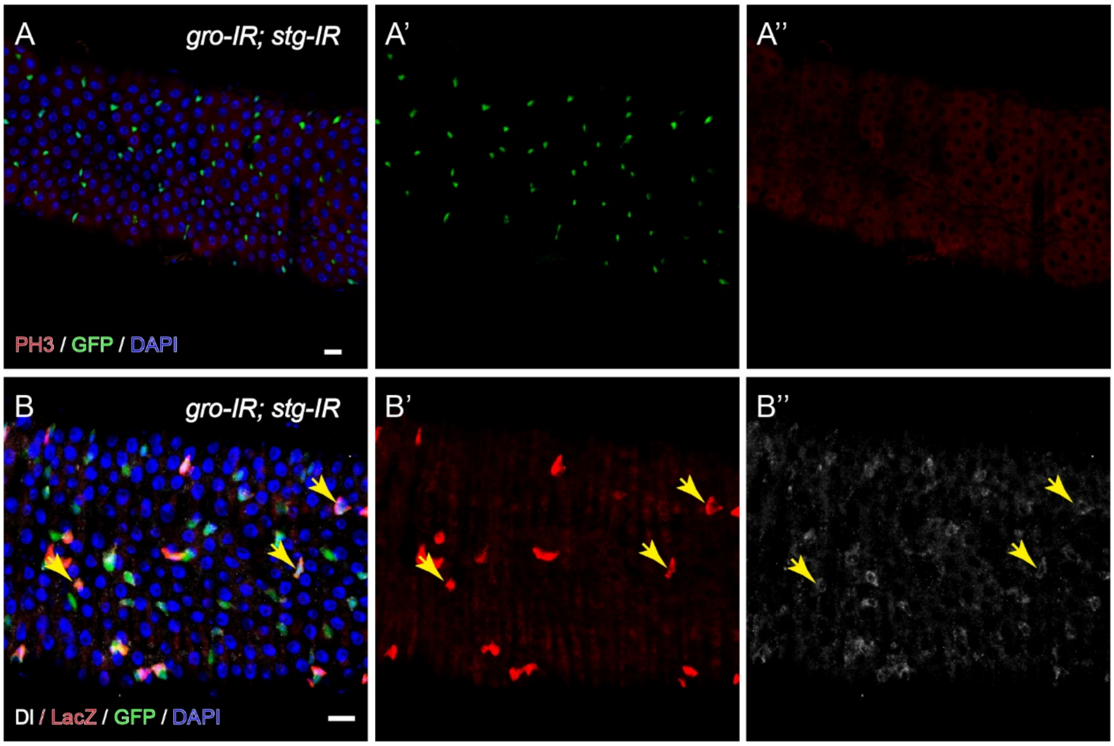

Figure S5, Related to Figure 2. Co-localization of Dl and NRE-lacZ signals could still be observed despite cell division was inhibited.

(A) Simultaneously knocking down *gro* and *stg* effectively reprerss *gro-IR* induced cell proliferation and PH3 signal is barely detected. (B) Dl^{+} NRE-$lacZ^{+}$ cells (yellow arrows) still exist in *gro-IR; stg-IR* guts.

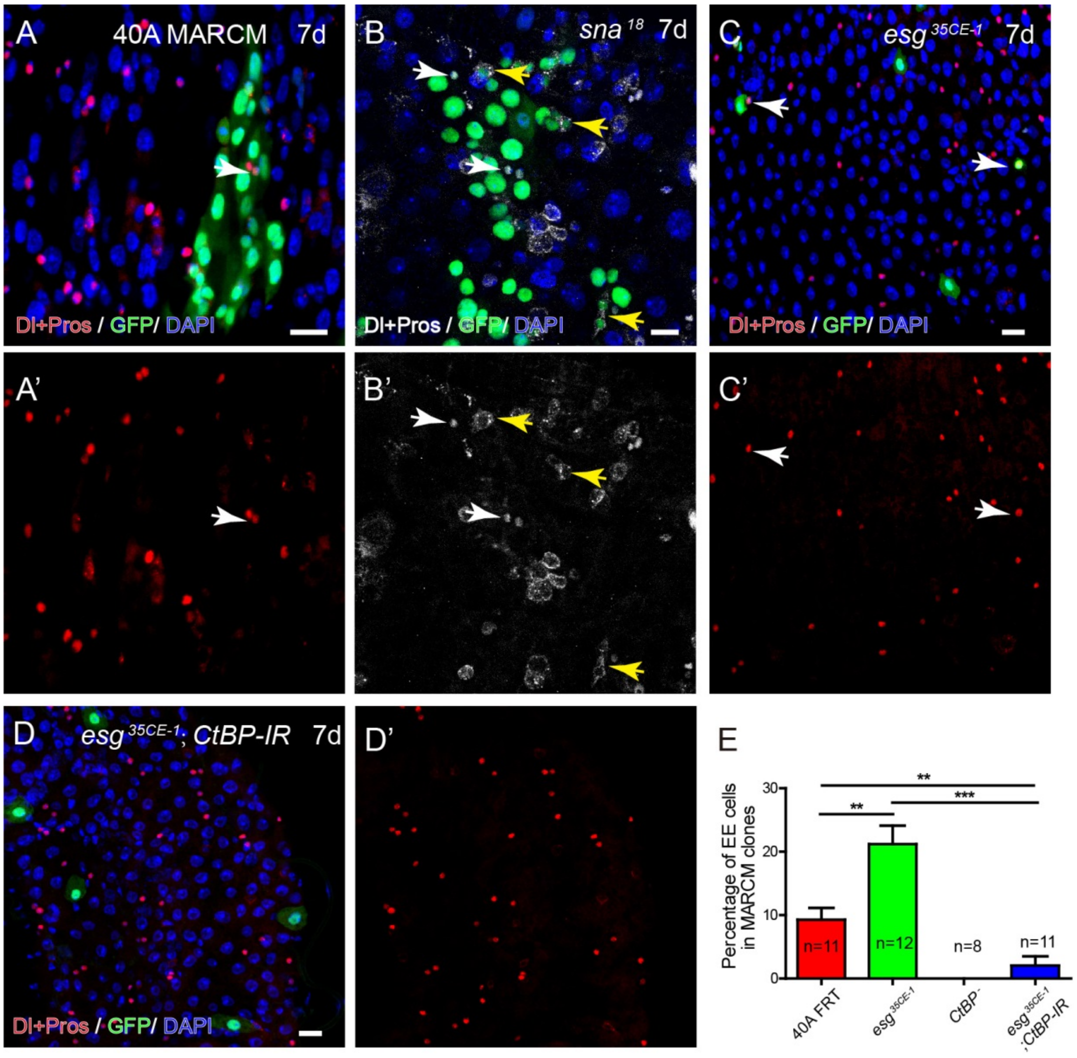

Figure S6, Related to Figure 4. Comparative analysis of *sna* and *esg* mutant clones and *esg CtBP* double mutant clones.

(A-B) Compared to control clones, depletion of *sna* exhibits no significant phenotype changes. Dl^+ cells are indicated by yellow arrows and $Pros^+$ EEs by white arrows. **(C)** *esg* mutant clones do not grow and cells in the clone can different into EC or EE. **(D)** Knocking down *CtBP* in *esg* mutant clones inhibits EE differentiation. **(E)** Quantification of EE percentage in MARCM clones. Error bars indicate mean ± s.e.m. The number of intestines calculated is labeled on the columns. **P<0.01 (Student's t-test).

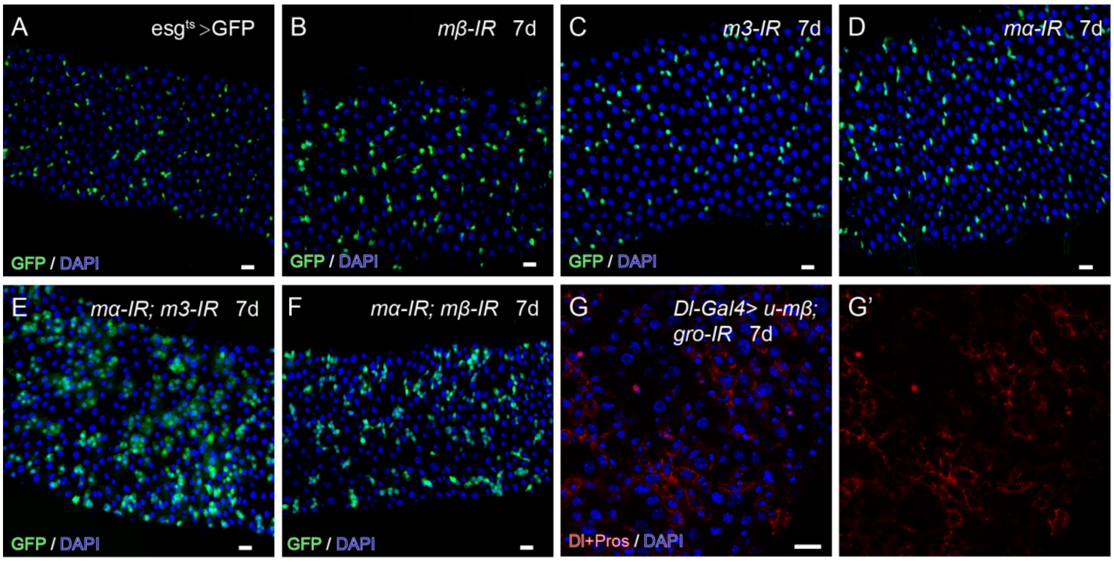

Figure S7, Related to Figure 6. *E(spl)* factors function redundantly to restrict ISC proliferation.

(A-D) Compared with control guts, separately knocking down *mα*, *m3* or *mβ* could not induce any obvious phenotype. **(E-F) S**imultaneously knocking down *mα* with *m3* or *mα* with *mβ* induced moderate accumulation of progenitor cells. **(G)** Knocking down *gro* rescues *mβ* overexpression induced ISC depletion, and causes accumulation of Dl^+ cells.
